# A poxvirus model reveals general correlates of antigen presentation and immunogenicity for viral CD8^+^ T cell epitopes

**DOI:** 10.1126/sciadv.aea8105

**Published:** 2025-12-19

**Authors:** Matthew J. Witney, Nathan P. Croft, Yik Chun Wong, Stewart Smith, Inge E.A. Flesch, Erica Keller, Leon CW Lin, Yanli Li, Nicole L. La Gruta, Anthony W. Purcell, David C. Tscharke

**Affiliations:** ^1^ John Curtin School of Medical Research, The Australian National University, Canberra, ACT 2601, Australia.; ^2^ Immunity Program and Department of Biochemistry and Molecular Biology, Biomedicine Discovery Institute, Monash University, Clayton, Victoria 3800, Australia.; ^3^ Institute of Biomedical Sciences, Academia Sinica, Taipei 11529, Taiwan.

## Abstract

CD8^+^ T cells are essential effectors in antiviral immunity that kill infected cells displaying viral peptide epitopes on major histocompatibility complex class I (MHC I). The pathways underpinning antigen presentation on MHC I are well known, but we lack a quantitative understanding of the relationships between source proteins and presented epitopes and how these relate to immunogenicity. We used mass spectrometry to interrogate vaccinia virus infection to reveal that up to 90% of epitopes were presented as fast as their source proteins were translated, but that protein amounts failed to correlate with epitope levels. Unexpectedly, epitope levels on infected cells also failed to correlate with immunogenicity. However, by extending our analysis to produce the first measurements of viral epitope levels from infected mice, we found a significant but moderate correlation with immunogenicity. These data provide empirical evidence for and against several associations that to date have been assumed or are not well resolved.

## Teaser

The amount of viral epitope presented on MHC I in lymphoid tissue of infected mice is a correlate of CD8^+^ T cell immunogenicity.

## INTRODUCTION

CD8^+^ T cells are critical effectors during viral infection that detect and eradicate infected cells. This detection relies on the presentation of short peptides, typically 8 to 12 amino acids long, processed from viral proteins and presented by major histocompatibility complex class I (pMHCI) on infected cells for recognition by T cell receptors (TCRs) on CD8^+^ T cells (*1*). Although the individual components of the antigen processing and presentation machinery are well described, the primary source of presented peptides, the relationship between antigen abundance and pMHCI levels, and the extent to which these might differ across cell types remain contentious. In particular, although it is accepted that peptides may be derived either soon after translation, e.g., as defective ribosomal products (DRiPs) (*2*–*5*), or from the turnover of mature proteins [so called “retirees” (*6*)], the fraction of these presented during viral infection from each of these sources remains controversial (*7*, *8*). Similarly, it remains unclear whether the most abundant viral proteins are the source of the most highly presented peptides (*9*, *10*). Last, while some studies have found that presentation differs markedly between cell types, without data on a wide range of epitopes, it is unclear whether these differences are outliers or the norm (*11*, *12*).

Antigen presentation on MHC I is important in two contexts in antiviral responses, for priming of CD8^+^ T cells by dendritic cells (DCs) and for killing of infected cells. For priming, antigen presentation drives two related characteristics of antiviral CD8^+^ T cell responses: (i) Only a relatively small number of theoretically possible viral peptide MHC I complexes (pMHCIs) are found to elicit a response; (ii) across the peptides that do elicit a response (called epitopes), the size of the CD8^+^ T cell response differs markedly (*13*–*15*). Intuitively, these seem to be two sides of the same coin because there should be a set of variables that operate up to a threshold, beyond which a peptide is immunogenic, and then further to determine immunogenicity. However, these parameters intersect in complex ways and have differing biologically relevant ranges. Therefore, the most useful parameter/s for sifting the few immunogenic from the many possible but nonimmunogenic peptides might not be a strong correlate of immunogenicity for known epitopes. Identifying variables in antigen presentation that are strong enough to stand out as single correlates of immunogenicity has value because it identifies the key drivers of the magnitude of antiviral CD8^+^ T cell responses.

A factor that has long been assumed to have the status of a key driver of immunogenicity is the abundance of epitopes presented, especially when measured on the DCs (*14*). The largest body of data underpinning this idea are from indirect measures related to presentation, such as pMHCI affinity and stability, which have been considered to reflect epitope levels (*16*). However, there is now disagreement across studies about whether pMHCI affinity is indeed a direct correlate of immunogenicity, or is better thought of as providing a useful threshold to discriminate immunogenic from nonimmunogenic peptides (*10*, *17*, *18*). Another strand of evidence is from studies showing that increasing the amount of a single model epitope presented during infection increases the size of CD8^+^ T cell responses (*19*–*21*). However, these reductionist experiments cannot show whether abundance is a strong predictor of immunogenicity across epitopes. By contrast, direct measurement of abundance by immunopeptidomic methods offers the best approach. However, most studies of this type have investigated too few epitopes to allow for statistically meaningful conclusions and/or have not used strictly quantitative methods (*15*, *22*–*30*). In the largest strictly quantitative study to date, the abundance of 22 influenza A virus epitopes and epitope-specific CD8^+^ T cells was measured in a mouse model of infection (*10*). This found a statistically significant correlation between the abundance of epitopes on infected cells and peptide-specific CD8^+^ T cell responses (*10*). However, it is unclear how well these findings can be generalized, in part because around 85% of all CD8^+^ T cells in the response to influenza virus in mice can be accounted for by only six peptides, with two peptides contributing around two thirds of the total response, reducing actual complexity. Demonstrating this, in subsequent modeling using multiple parameters, a single dominant peptide determined the best correlate of immunogenicity (*10*).

A viral infection model in which there are a large number of well-defined epitopes that span wide ranges of abundance and immunogenicity would be ideal to comprehensively address questions about antigen presentation and immunogenicity. Vaccinia virus (VACV) has long been used as a tractable model pathogen for studying CD8^+^ T cell responses and has a large proteome (*31*). It is arguably the most celebrated of vaccines, having been used to eradicate smallpox, and is back in the limelight as the vaccine for Mpox. VACV has 170 mass spectrometry (MS)–validated H-2^b^ pMHCI for which immunogenicity has been rigorously tested (*15*, *30*) and mouse models of infection are strictly acute with presentation of epitopes on infected DC directly priming CD8^+^ T cells (*32*–*38*). Here, we have taken advantage of these features of VACV infection, collecting large MS datasets from four cell types at multiple times after infection and then going further to measure the abundance of epitope presentation ex vivo from infected mice. Relating these data to rigorous measurements of epitope-specific CD8^+^ T cell responses has allowed us to make robust conclusions about the factors that drive antigen presentation and immunogenicity in viral infection.

## RESULTS

### Abundance of 45 VACV pMHCI on cells infected in vitro

**Fig. 1.**
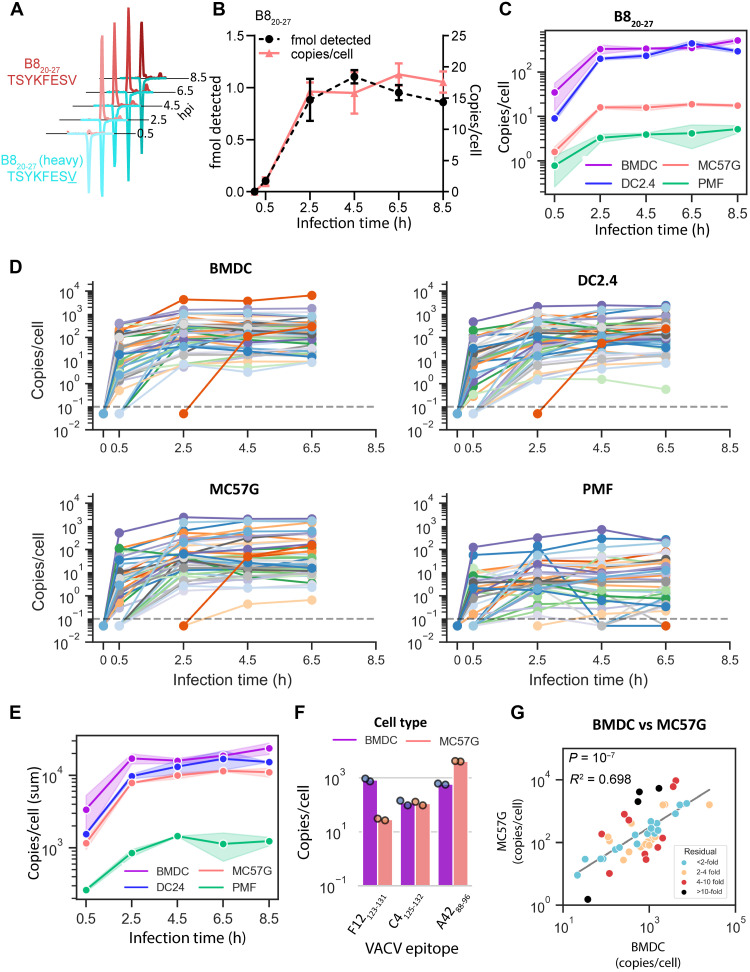
Quantification of 45 VACV epitope presentation on infected cells over time. Cells were infected in vitro with VACV, and epitope presentation was quantified by MRM MS at the times shown. (**A**) Chromatogram peaks representing detection of VACV-specific and isotopically labeled B8_20-27_ peptide presented on VACV-infected MC57G. hpi, hours post infection. (**B**) B8_20-27_ peptide detected by MS (black), or calculated B8_20-27_ peptide copies per cell (pink), at each time point on MC57G cells. (**C**) Abundance of B8_20-27_ presented on H-2K^b^ over time in BMDC, DC2.4, MC57G, and PMF cells. Shaded regions represent the range from two independent replicates. (**D**) Abundance of 45 VACV epitopes over time presented on BMDC, DC2.4, MC57G, and PMF cells. Lines represent the average abundance of an epitope from two independent replicates and are drawn from the time point prior to the time of first detection. Points below the dotted gray line identify undetected peptides. (**E**) The sum abundance of all 45 epitopes on each cell type at each time point. Shaded regions represent the range from two independent replicates. (**F**) The sum of three epitope abundances over time for three VACV epitopes presented on BMDC and MC57G cells. (**G**) The abundance of 45 VACV epitopes on BMDC and MC57G cells. Abundance is presented as the average sum of all time points for each epitope. Peptides are colored by the distance from the log-log regression line to highlight outliers. h, hours.

To measure the abundance of 45 pMHCI on VACV-infected cell types, we used the sensitive MS technique of multiple reaction monitoring (MRM) MS, using internal isotopically labeled standard peptides to provide accurate quantification of endogenously presented viral peptides (Fig. 1A and data S1). Cells were infected in vitro with VACV between 0.5 and 8.5 hours to allow time for the complete VACV replication cycle (*4*). The absolute abundance of peptide detected by MS can be normalized to the number of cells, providing a measure of epitope density per cell (Fig. 1B). Using this strategy, the abundance of 45 VACV epitopes was measured on bone-marrow–derived dendritic cells (BMDCs), primary murine fibroblasts (PMFs), as well as the dendritic-like and fibroblastic-like cell lines, DC2.4 and MC57G, respectively (Fig. 1, C and D, and data S1) (*39*, *40*).

Nearly all epitopes were detected within 2.5 hours after infection; however, the range of epitope levels observed on each cell line varied greatly and could differ by more than 1000-fold at a given time point (Fig. 1D). At each time point, total epitope levels, represented by the sum abundance of all 45 pMHCI, were the highest on BMDCs. The most notable difference was the 10-fold lower total epitope presentation on PMFs compared to the other cell types (Fig. 1E).

To identify whether cell type affected how well individual epitopes were presented, we compared the sum abundance of each epitope in each time course between all four cell types. For individual epitopes presented on the most different (primary DC versus immortalized fibroblast) BMDCs and MC57G cells, we could identify examples where the absolute abundance of an individual epitope had higher, the same, or lower levels on either cell type (Fig. 1F). However, from a multi-epitope perspective and when displayed on a log_10_ scale, the relative abundance of all 45 epitopes on BMDCs and MC57G cells was highly significantly correlated (Fig. 1G). Comparing individual epitope levels between all pairs of cell types, the dendritic-like BMDCs and DC2.4 cells were the most closely related (fig. S1A). Taking this further, epitopes that were more abundant on primary BMDCs compared to PMFs were also more abundant than expected on immortalized DC-like DC2.4 compared to fibroblastic MC57G cells (fig. S1, B and C). This suggested that similarities in cell type of origin could partially explain how well an individual epitope was presented. A similar comparison between epitopes presented on immortalized cells compared with primary cells was also significant, but the association was not as strong (fig. S1D). Last, when a principal components analysis (PCA) was done to examine all cell types and all times, the major contributor to differences as seen in principal component 1 (PC1) and PC2 was overall abundance, which separated cell types and time after infection, respectively (fig. S2).

We concluded that VACV pMHCI abundance on different cell types in vitro was generally well correlated and, at the same time, noting that cell type was the main driver of differences and that for some individual epitopes, differences can be notable.

### Abundance of mouse and VACV proteins in infected cells in vitro

The changes in epitope levels between cell types could be driven by differential antigen presentation machinery expression or efficiency, or altered VACV protein expression. To address this, we quantified the abundance of viral and mouse proteins from the same set of VACV-infected cell samples using a label-free quantification (LFQ) MS method. LFQ provides a measure of the relative abundance of proteins, without requiring additional protein standards (*41*). A total of 151 VACV proteins were detected across the infected cell types, accounting for ~70% of the proteins encoded in the VACV genome (fig. S3). Simultaneously, 3588 mouse proteins were detected, and their abundance was estimated (data S1).

**Fig. 2.**
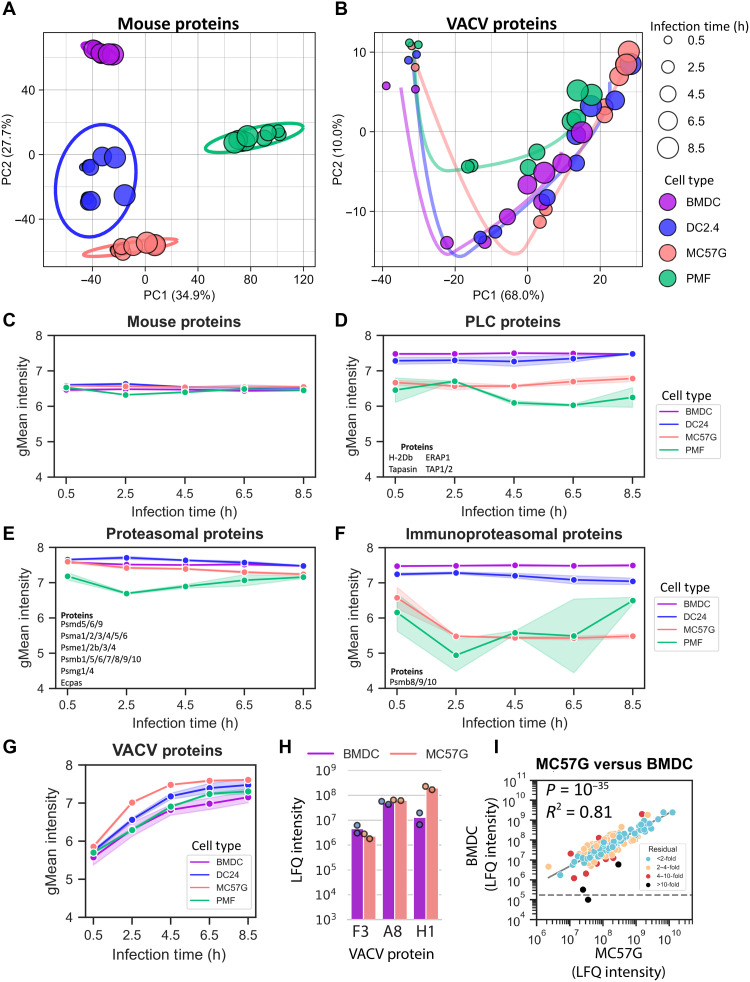
LFQ of mouse and virus proteins in vitro on VACV-infected cells. BMDC, DC2.4, MC57G, and PMF cells were infected with VACV, and protein abundance was estimated by LFQ. PCA of mouse (**A**) and VACV (**B**) protein abundance. Clusters identify samples from common cell types using PC1 and PC2 (A) or trace a trajectory of PC1 and PC2 with respect to time projected onto the PC1-PC2 axis (B). (**C** to **G**) The geometric mean (gMean) of protein LFQ intensity per replicate at each time point and cell type for all mouse proteins (C), proteins associated with the peptide loading complex (PLC) (D), proteasomal degradation (E), immunoproteasome subunits (F), and all VACV proteins (G) LFQ intensities for each cell type at each time point. Shaded regions draw the range of values from two independent replicates. (**H**) The sum of protein LFQ intensities over the infection time course for three selected VACV proteins. (**I**) Correlation of VACV protein abundance between MC57G cells and BMDCs. VACV protein abundance was calculated as the average sum of all time points for each protein on log-transformed values. Proteins are color coded by fold distance from the log-log linear regression line. Points below the horizontal dashed line identify proteins not detected.

PCA was used to explore protein abundance in this dataset, either examining the mouse and virus proteomes separately (Fig. 2, A and B) or combining them (fig. S4). Mouse proteins alone clearly clustered samples by cell type rather than infection time, suggesting that changes in the mouse proteome due to infection were minor compared to the differences between cell types (Fig. 2A). The stability of the host proteome and the massive abundance of the relatively small number of viral proteins are evident when comparing total amounts of protein with the amounts per protein between host and viral proteomes (fig. S5). Variability in the abundance of VACV proteins was best explained by shifts in protein abundance over time represented by samples following a similar trajectory on the first two PCs regardless of cell type (Fig. 2B). The PCA loadings of each protein suggest that PC1 largely represented an increase in the abundance of a VACV protein as the virus replication cycle progressed. PC1 and PC2 also separated classes of VACV genes with similar transcriptional kinetics during the virus replication cycle, which can also be seen reflected in a similar analysis of presented epitopes (fig. S6) (*4*, *42*, *43*).

In addressing potential differences in antigen presentation efficiency across cell types, we found that PMFs and MC57G cells had a lower abundance of proteins associated with pMHCI processing and presentation, and these varied more than the mouse proteome as a whole (Fig. 2, C to F). Notably, PMFs and MC57G cells had very low immunoproteasome content and relatively low abundance of proteins that directly form the peptide loading complex in the endoplasmic reticulum. These findings support the use of these four cell types as representing professional and nonprofessional antigen-presenting cells (APCs).

Compared with the differences seen for antigen presentation machinery between DCs and non-DC cells, total VACV protein abundance on these cells was more comparable across cell types (Fig. 2G). Looking at individual viral proteins, while some individual proteins were found to have higher or lower abundance across the different cell types (Fig. 2H), overall, the correlation of abundance of VACV proteins between cell types was very tight (Fig. 2I). Notably, the correlations of viral proteomes between all pairs of cells were closer than was seen for epitope abundance (fig. S7).

Taking all these data together suggests that poor levels of antigen presentation machinery rather than poor infection or expression of viral proteins explain the low levels of VACV epitopes on PMFs and differences in presentation across cell types. The importance of antigen presentation efficiency is further underlined by the results from infected BMDCs, which had the highest levels of epitope presentation, yet the lowest levels of VACV proteins at each time after infection.

### Most viral epitopes are presented immediately after translation

**Fig. 3.**
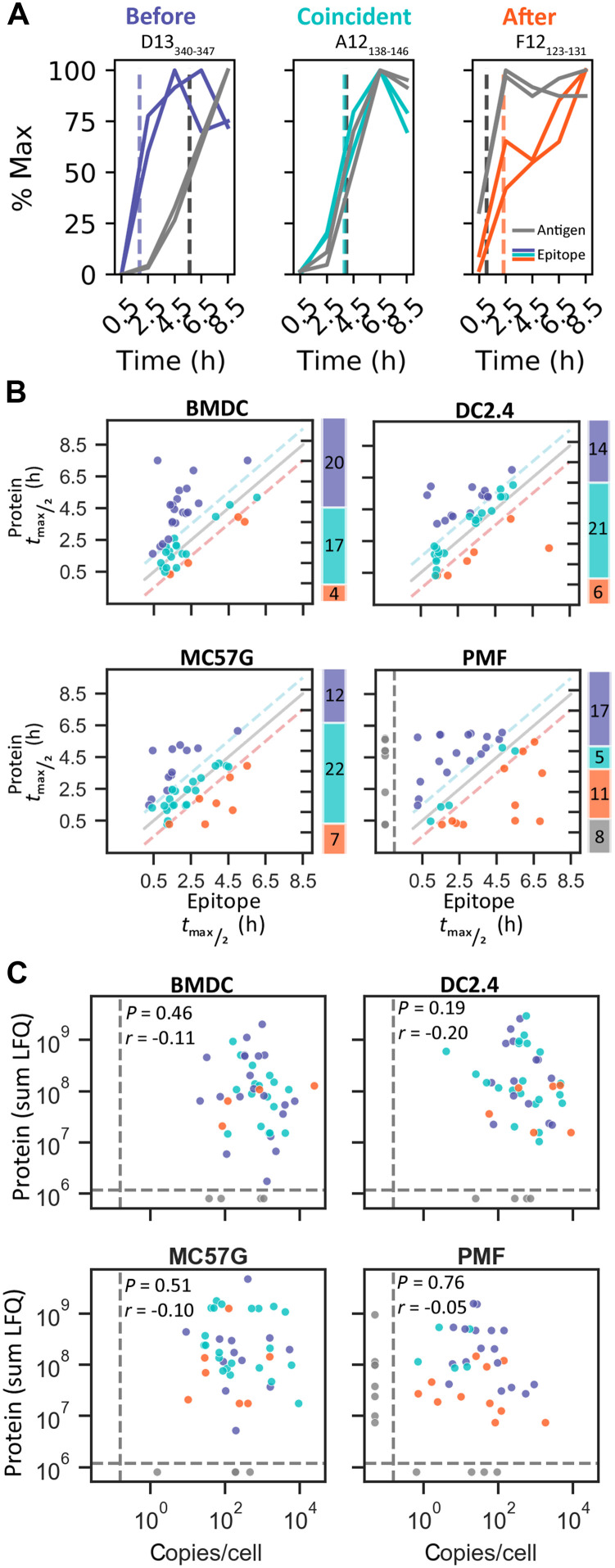
VACV epitope presentation predominantly occurs coincident with translation and does not correlate with source protein abundance. Epitope and protein abundance were normalized to the maximum value in each replicate over the infection time course. Four VACV epitopes with undetectable protein levels are excluded from analysis in this figure. (**A**) Representative VACV epitopes on infected BMDCs that show epitope time to half-maximum levels (*t*_max/2_) occurring before, coincident with, or after the respective protein. The two lines for epitope and protein show the duplicate measurements. Dotted lines identify the mean time to *t*_max/2_ for epitopes and protein. (**B**) Comparison of the time to *t*_max/2_ for matched epitope and antigen. Diagonal lines identify values representing identical epitope and protein half-life or ±1 hour, used to classify epitope kinetics as before (blue), coincident with (aqua), or after (orange) protein expression or undetected epitope (gray). Bar charts show the number of peptides classified into each category. (**C**) Comparison of the sum of each epitope and protein abundance (LFQ) over time on VACV-infected cell types. Statistics: Spearman correlation.

To investigate the relationship between epitope presentation and antigen expression kinetics, we compared the absolute abundance of VACV epitopes and the relative abundance of their source proteins at each time point. Previous comparisons of this kind have used a handful of proteins and made visual comparisons comparing the time of peak expression (*10*, *30*). Here, our comparisons were done using 41 epitope/source protein pairs (we did not detect all viral proteins). Further, to make a formal quantitative comparison, we calculated the time taken for each epitope and protein to reach the half-maximal levels detected across the time course. Epitopes were classified according to whether epitope half-maximal levels were reached an hour or more earlier (before), within an hour (coincident), or an hour or more later (after) the protein half-maximal levels (Fig. 3, A and B, and fig. S8). Most epitopes reached half-maximal levels either coincident with or before the respective source protein. This was particularly evident on infected BMDCs, where nearly all epitopes had achieved half-maximal levels by 2.5 hours of infection, and 37 of 41 epitopes (>90%) were classified as reaching half-maximal levels before or coincident with their source protein (Fig. 3B). However, it was notable that in non-DC cell types and especially on the PMFs, more epitopes appeared later and reached half-maximal levels after their source virus proteins. These results suggest that there are functions of professional APCs that ensure that most viral proteins are not only processed efficiently but also soon after translation, if not cotranslationally.

The time to reach half-maximal levels for epitopes derived from the same source viral protein appeared to be more closely related than for unrelated epitopes (fig. S9), although we could also identify exceptions to this trend. For example, two epitopes derived from the VACV protein A47 were presented either “before” or “after” the detection of the mature antigen. This shows that the same protein can enter the antigen presentation pathway at more than one point over its life span. Likewise, across cell types, the same epitope could be classified into different kinetic classes. These transitions were generally between the “before” and “coincident” classes, suggesting that any cell type–specific changes were mechanistically conservative (fig. S9). However, we also noted a potential polarization of the epitope kinetics on PMFs into either “early” or “late,” classification, which is what drove the increase in the number of epitopes appearing after their source protein on PMFs (Fig. 3B).

### Epitope abundance does not correlate with source protein amount or affinity for MHC I

Next, we investigated whether the epitope presentation and respective VACV source antigen levels are related during VACV infection. Across this large set of epitopes and multiple times after infection on four different cell types, there was no correlation between protein and epitope abundance (Fig. 3C). This was irrespective of the kinetic relationship between proteins and epitope explored above. These data show definitively that relative amounts of proteins do not predict levels of epitope presentation across a viral proteome. The binding affinity of pMHCI has often been thought to be a major determinant of presentation levels. We tested this with our data using previously published affinities (*15*) and found that irrespective of cell type, the level of presentation was not significantly correlated with binding affinity of the epitopes for their presenting MHC allomorph (fig. S10A).

### Epitope abundance in vitro does not correlate with CD8^+^ T cell responses

**Fig. 4.**
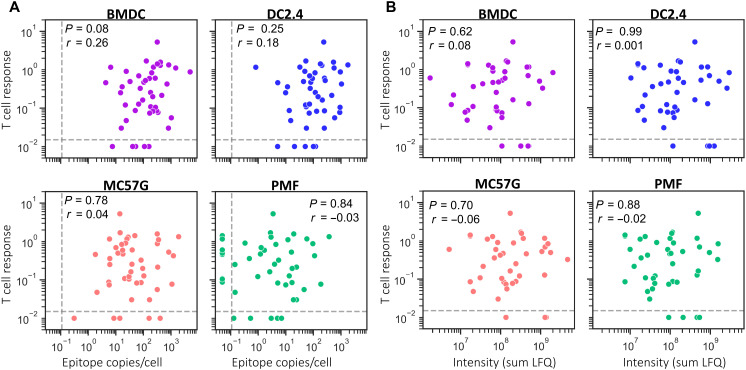
Epitope and protein abundance in vitro do not correlate with the size of peptide-specific CD8^+^ T cell responses. Epitope (**A**) and protein (**B**) abundance were calculated as the average of the sum of values over the time-course from two independent replicates. CD8^+^ T cell responses measured in the spleens of mice 7 days after intraperitoneal infection of C57BL/6 mice were from Dataset 1 in Croft *et al.* (*15*). Statistics: Spearman correlation.

Epitope abundance is often suggested to be a primary factor contributing toward the size of peptide-specific CD8^+^ T cell responses during virus infection (*20*, *21*). Using the complete set of 45 epitopes, we correlated epitope abundance with immunogenicity during acute intraperitoneal VACV infection in C57BL/6 mice using data published previously (*15*). The size of peptide-specific CD8^+^ T cell responses did not correlate with epitope abundance measured on any cell type (Fig. 4A). A possible caveat to this conclusion might be if the peak of CD8^+^ T cell responses to VACV is sharp and varies considerably across epitopes, in which case we would not be correlating abundance of presentation with the maximal response to each epitope. However, using a selection of epitopes, we found that all responses rose from day 6 to day 7 and thereafter remained at a similar level until day 9 (fig. S10B). Having found that epitope abundance did not correlate with immunogenicity, for completeness, we looked to see whether there was a relationship between the relative amounts of viral proteins measured in vitro and T cell responses and found that viral protein levels and T cell responses were not correlated (Fig. 4B).

### In vivo quantification of epitope abundance and matched CD8^+^ T cell responses

**Fig. 5.**
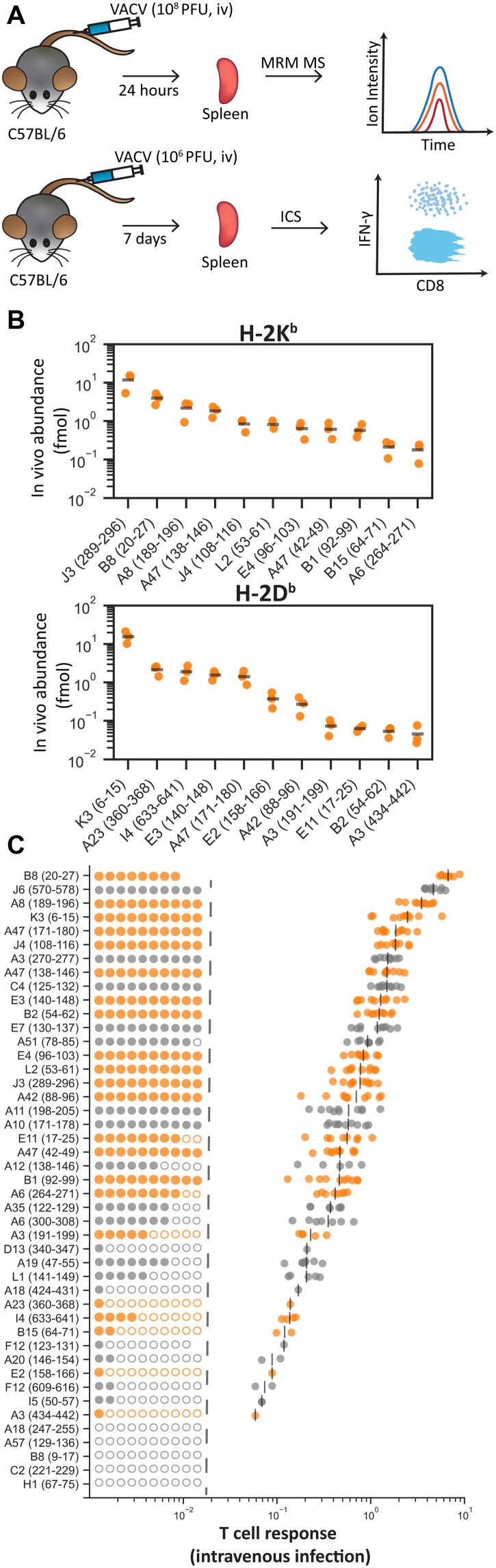
A shared experimental model for in vivo quantification of epitope abundance and measurement of CD8^+^ T cell responses. (**A**) Schematic showing the matched experiments to quantify peptides ex vivo and measure peptide-specific CD8^+^ T cell responses. (**B**) Abundance of VACV peptides presented on H-2K^b^ and H-2D^b^ on splenocytes 24 hours after intravenous infection with VACV; *n* = 3. Peptides not detected in any mouse are not shown. (**C**) CD8^+^ T cell responses to 45 VACV peptides in the spleen 7 days after intravenous (iv) infection. Each peptide was tested in 8 to 10 mice. Peptides detected by MRM MS in the spleen as in (B) are colored orange. Filled and empty circles left of the dashed line represent mice in which each peptide was immunogenic (filled) or nonimmunogenic (empty). The size of responses is shown on the right, with a black bar showing the mean.

The experiments above were done based on the premise that the actual APCs in vivo would display epitopes in a similar fashion to at least one of the cell types in our in vitro experiments. Looking more closely at the cell-type differences in epitope abundance, we noted an incremental improvement in the correlation coefficient between epitope abundance and T cell responses from fibroblastic cells to those representative of a more likely APC in vivo, that is, presentation level on BMDC showed a higher Spearman correlation with CD8^+^ T cell magnitude than that on DC2.4, which in turn was higher than seen for MC57G and PMF. For this reason, a new strategy was devised in which we set out to measure VACV epitope abundance in vivo in an experimental infection model that we could match for the measurement of CD8^+^ T cell responses. In vivo (or direct ex vivo) measurement of multiple viral epitopes during infection has not been achieved previously, so we used a high dose of virus delivered intravenously and examined spleens after 24 hours, when priming should be occurring. The second part of the experiment was to measure CD8^+^ T cell responses to VACV epitopes after an intravenous infection to account for any difference in immunodominance by route of infection (Fig. 5A) (*37*).

Using MRM MS to measure the abundance of each epitope, 22 of the 45 epitopes investigated in this study were detected in the spleen after infection, spanning over a 300-fold range in abundance (Fig. 5B and data S1). To determine whether the detection or otherwise of peptides ex vivo might be linked to other characteristics, we compared the (i) abundance of presentation on BMDC, (ii) the measured peptide affinity for MHC, (iii) the size of CD8^+^ T cell responses, and (iv) the kinetic class, for the epitopes detected, not detected, and the full group of 45 (fig. S10C). Of these, only the size of CD8^+^ T cell responses differed significantly between peptides detected and not detected, perhaps suggesting that our detection of presented peptides was an indication of immunogenicity. However, none were significant when comparing the 22 peptides detected in vivo against the original full set of 45. Further, the failure to find a significant correlation between abundance and affinity for MHC seen for infected cells in vitro was also seen for abundance measured in vivo (fig. S10D). Although not significant in our numerical analysis, it was curious that when ranked by abundance, the top two thirds of peptides found in the spleen were all from early genes and none of the detected epitopes were from late genes, suggesting a preference for epitopes from proteins expressed early during infection to be detected in vivo. Overall, we concluded that the 22 epitopes detected in the spleen spanned a similar range in all important parameters as the initial group of 45 peptides and were therefore representative.

Next, the magnitude of peptide-specific CD8^+^ T cell responses in the spleen 7 days after intravenous infection with VACV was measured by restimulation of splenocytes with peptides followed by staining for intracellular interferon-γ (IFN-γ ICS; Fig. 5C, fig. S11, and data S1). This is the method most commonly used to measure peak CD8^+^ T cell responses to multiple epitopes after VACV infection, including after intravenous infection, and gives results that are similar to pMHC tetramers (*15*, *21*, *30*, *37*, *44*, *45*). Consistent with previous virus infection models, some peptide-specific CD8^+^ T cell responses were detected in a fraction of mice (*10*, *15*). In these cases, the average peptide-specific responses were calculated only from mice where an immunogenic peptide-specific response was detected. These results highlight the diversity of the CD8^+^ T cell response to VACV, which spans over two orders of magnitude (6.6 to 0.01% of CD8^+^ T cells) and with the most immunogenic epitope contributing 17% of the total and >20 epitopes each contributing more than 1% of the total response (fig. S11).

### Epitope levels in vivo are a moderate correlate of CD8^+^ T cell responses

**Fig. 6.**
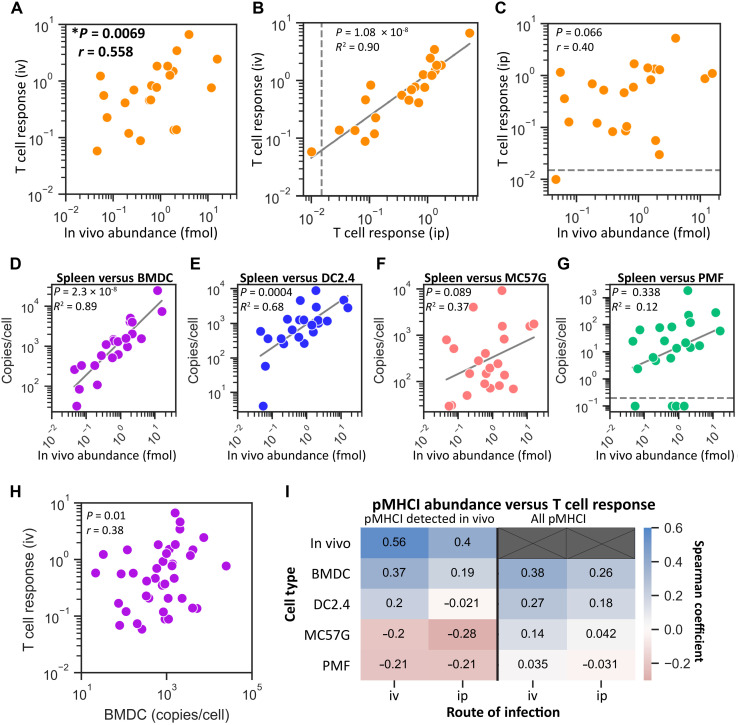
In vivo measurement of epitope abundance correlates with peptide-specific CD8^+^ T cell responses following intravenous infection. (**A**) Correlation between the average epitope abundance in vivo and the average immunogenic peptide-specific CD8^+^ T cell responses following intravenous infection with VACV (data from Fig. 5). (**B**) Comparison of peptide-specific CD8^+^ T cell responses in C57BL/6 mice following intraperitoneal (ip) and intravenous infection. (**C**) Comparison of peptide-specific CD8^+^ T cell responses following intraperitoneal T cell responses in C57BL/6 mice and epitope abundance measured in vivo. (**D** to **G**) Correlation between epitope levels measured in vivo and on VACV-infected cells (as noted) in vitro. (**H**) Comparison of epitope abundance measured on infected BMDCs in vitro and peptide-specific CD8^+^ T cell responses following intravenous infection. (**I**) Degree of correlation between epitope abundance and peptide-specific CD8^+^ T cell responses. Comparisons include the 22 pMHCI detected in vivo or the complete set of 45 epitopes (all pMHCI). Statistics: Spearman correlation (A, B, H, and I); Pearson correlation (C to G).

Using the set of 22 epitopes detected in vivo, we examined the relationship between epitope abundance and the size of peptide-specific CD8^+^ T cell responses following intravenous infection. We now identified a significant, albeit moderate, correlation between epitope abundance in vivo and peptide-specific CD8^+^ T cell responses during VACV infection (Fig. 6A).

To understand why we identified a statistically significant correlation between T cell response and epitope abundance in this final experiment, we asked whether this was primarily associated with a change in T cell response between the two routes of infection, or by the measurement of epitopes in vitro versus in vivo or a combination of both. The CD8^+^ T cell immunodominance hierarchies determined via intraperitoneal and intravenous infection were very closely correlated (*R*^2^ = 0.90) (Fig. 6B). However, despite the difference in responses for epitopes being apparently small between these two routes, it was enough to undermine the significance of the correlation between response and epitope abundance (Fig. 6C). Likewise, epitope abundance in vivo and on cells in vitro was very strongly correlated for BMDCs and DC2.4 infected cells in vitro. However, this correlation was not significant for MC57G and PMF (Fig. 6, D to G). The close relationship between epitope levels measured in vivo and those from infected DC in vitro is consistent with previous work showing VACV in DCs and macrophages in the lymph nodes and spleen of infected mice (*46*–*50*). However, these studies looked earlier after infection, and the spleen is a complex tissue with many cell types, so the cellular source of the peptides we detected here cannot be determined without further experimentation. The relationship between presentation on BMDC and in the spleen in vivo was so close that we re-examined whether the epitope amounts presented on BMDC might correlate with the T cell response after intravenous infection. This was the case when we included all 45 epitopes measured in vitro (Fig. 6H). However, a summary of the correlation coefficients for all combinations of epitope abundance and CD8^+^ T cell measurements shows how quickly the association between these two parameters degrades once they are no longer matched (Fig. 6I). As a final analysis, we asked whether the time that epitope levels were quantified after infection in vitro might be more accurate than summing presentation (fig. S12). We found that epitope levels measured at 2.5 hours after infection of DC cell types were the best correlate of immunogenicity and were superior to the sum of presented amounts (*r* = 0.42 versus 0.38 for BMDC) but remained weaker than ex vivo quantification.

Together, these observations demonstrate that levels of epitope presentation are a moderate correlate of the size of T cell responses; however, the relationship is sufficiently weak that it is easily obscured by other variables. As a result, the significance of the correlation only emerges if confounding variables are reduced by very close matching of the models used to measure epitope abundance and T cell responses and/or a very large number of epitopes is examined.

## DISCUSSION

Here, we set out to address four fundamental questions about antiviral immunity: (i) the extent to which presentation of epitopes differs across cell types, (ii) whether antigen levels predict epitope levels, (iii) the kinetic relationship between viral protein production and epitope presentation, and last, (iv) whether epitope presentation levels are a significant driver of immunogenicity. We were seeking answers to these questions across a wide range of epitopes, rather than whether these things might be true for a single epitope. Answers to these questions have been attempted previously, but the literature is inconsistent, due mainly to studies not having enough epitopes in an inherently noisy system but also because absolute quantification of presentation has been rare (*10*).

First, we found that viral epitope presentation levels spanned orders of magnitude and broadly correlated between all infected cell types in vitro (Fig. 1 and fig. S1). This was most notable for PMFs, in which epitope abundance correlated with abundance from all other cell types despite the globally lower presentation levels of all epitopes. Having noted this overall result, individual epitopes could be found that differed in abundance by more than 10-fold between different cell types. This helps to explain disparate findings in the literature where fewer peptides were examined and is reminiscent of data published for influenza A virus, though the correlation shown here for VACV epitopes was stronger (*10*). The differences we observe are most likely related to antigen processing rather than in virus infection, because the correlation of viral protein levels between pairs of cells was higher than for epitopes (Fig. 2). Again, PMFs provide a case in point: We found similar levels of viral protein abundance in these cells compared with the other cell types but low levels of antigen processing and presentation machinery, presumably leading to the poor levels of epitope presentation (fig. S6). More subtle shifts in the relative abundance of epitopes, such as between BMDCs and MC57G cells, could arise from the altered biases in antigen processing, for example, due to immunoproteasomes (in BMDCs) compared to constitutive proteasomes (in MC57G) (*51*, *52*). The fairly subtle changes we saw associated with proteasome type is more consistent with another systematic, quantitative analysis (*53*) but not previous studies that aimed to identify the peptides presented (*51*, *54*, *55*). However, a caveat is that we are using cells that will express a natural range of proteasome types, not testing the effect of a complete absence of immunoproteasome subunits (*51*, *54*, *55*), and it remains unknown the extent to which proteomes might be affected by virus infection.

Second, the expression kinetics and levels of viral proteins were not obviously reflected in these parameters for their epitopes. Therefore, it was not unexpected that, in our experiments, source protein amounts failed to correlate with the abundance of epitopes, a finding that held at all times after infection and for all cell types. This is consistent with similar experiments using influenza virus and where the self-proteome was disturbed by treatment with interferon (*10*, *55*). However, it contradicts other studies of homeostatic self-antigen presentation (*56*–*59*). This might represent a difference in context between viral or induced protein expression versus homeostatic expression of self-antigens, but may also reflect different methodologies: We are directly measuring protein levels where other studies have inferred abundance from the likelihood of identification or RNA sequencing data (*56*, *58*, *59*). There is also new evidence that the quality control of translation is responsible for the presentation of many self-peptides on MHC I (*60*). Irrespective of any difference between viral and self-peptides, from the large set of epitopes and proteins we measure here, it seems unlikely that protein abundance is going to be found to be a useful predictor of epitope presentation levels for other viruses.

Third, as suggested in studies of influenza virus and Epstein-Barr virus, we find here that most VACV epitopes are processed from their source protein as quickly as these proteins are translated (*10*, *61*). This and the lack of correlation between viral protein and epitope levels noted above suggest that DRiPs and similar products of translation are the main source of viral epitopes, with retirees making a minor contribution. Adding some nuance to our finding is the extent to which this occurs in professional APCs and fibroblasts, with the difference most notable between the primary cells. In the BMDCs, less than a tenth of the source proteins reached their half-maximal level before the epitope, but for PMFs, this is a third. It is tempting to speculate that the specialization of DCs for presentation goes beyond the immunoproteasome and superior levels of protein components of the antigen processing machinery and extends to an ability to sample newly translated proteins more effectively. Why some epitopes are not rapidly presented coincident with translation of the source antigen is not clear, although this may be associated with the rate of protein translation, or turnover as has been suggested for self-peptides (*58*). In favor of the idea that it is a property of the viral protein, where we have epitopes derived from the same source antigen, they tend to have a similar epitope:antigen kinetic profile, and this is maintained across cell types. A limitation of our analysis and data is that we measure only bona fide proteins, and this means we have not been able to detect very short-lived or noncanonical products of translation that may be sources of epitopes. It remains a challenge in the field to catch the natural DRiPs from an infection model to understand their characteristics and relationship to antigen presentation more directly.

Last, we have assessed the extent to which the abundance of viral epitope presentation is correlated with immunogenicity. This is another area where previous studies are split between finding correlations or not, across a range of viruses and number of epitopes under investigation (*10*, *24*, *27*, *28*, *30*). The contribution that we make here is twofold: (i) We answer this question with a larger number of epitopes such that a moderate correlation could be demonstrated, and (ii) by making multiple measurements of epitope abundance in vitro and in vivo, we show why it has been difficult to come to a consensus on what should be a fundamental question. The difficulty in identifying direct correlates of immunogenicity is not unexpected given the complexity of the biology, myriad sources of noise, and the relatively minor contribution made by individual parameters. Beyond antigen presentation, epitope-specific T cell responses are subject to multiple factors associated with the responding T cells, including the number of naïve T cell precursors, TCR affinity for an epitope, and susceptibility to immunodomination (*17*, *27*, *62*–*65*). These factors will have a significant role in shaping immunogenicity. We speculate that epitope affinity for MHC I might have an unappreciated role in stabilizing peptide-MHC-TCR complexes as has been suggested previously (*10*). We did not find a correlation between affinity for MHC and either epitope presentation levels or size of T cell responses, despite affinity being the best predictor of whether a given peptide might be immunogenic (*10*, *15*, *17*). The caveat here is that we use a set of peptides with a restricted range of affinities. However, it remains the case that presentation levels varied over three to four orders of magnitude, so if peptide affinity were correlated, as opposed to being a thresholding variable, we would have expected this to be observable in our data.

The variability in the literature is likely to derive from studies with too few epitopes, but also with differences in how epitope abundance was measured, including cell and infection type and how closely this mimics presentation in vivo on the DCs that prime the CD8^+^ T cell response. For VACV, we show a sequence of shifts in presentation amounts looking across our data from in vivo at one end to infected fibroblasts at the other, and differences in immune responses to intraperitoneal and intravenous infection. Had these studies been published separately, we might have interpreted our results in a study of presentation on fibroblasts as finding no correlation between abundance of epitope and T cell responses, and then the opposite in a second study in which we measured abundance in vivo. Presenting these together here makes it clear that the association is somewhat fragile so it matters how and where epitope abundance is measured, with in vivo being the most appropriate.

The best infection model for understanding antigen presentation and predicting immunogenicity depends on the virus or other pathogen used. For VACV, there is substantial evidence that infection of DCs and direct presentation of epitopes during VACV infection are the dominant mode of priming for most CD8^+^ T cells (*16*, *21*, *34*, *46*, *48*, *66*). This presents an apparently simple model to interrogate the relationship between epitope level and CD8^+^ T cell responses. However, further work is required to be sure that this model is sound: First, epitope presentation should be measured at multiple times after infection; second, the site and identity of infected cells must be shown, and we need to know if these cells are in fact the source of viral peptides detected by MS; last, virus doses used in epitope measurement should be reduced to match the dose used to measure T cell responses. Even with these things done, we would remain a long way off the ideal viral model, which would comprise only the correct subset of DCs infected under conditions that mimic natural infection. Using whole spleens and BMDC preparations that are not homogenous will have introduced multiple sources of variation. Last, moving beyond VACV, there is still a need to interrogate other models where the infection is chronic or where cross priming is important, as in the case of influenza virus (*67*). Nevertheless, we would expect that the general link between epitope abundance and CD8^+^ T cell responses shown here is likely to hold for other virus infections.

Overall, we demonstrate the value of VACV as a model to sift general correlates of antiviral T cell responses from the complexity of factors that come together in antigen presentation and priming. We find that the vast majority of viral epitopes are presented as soon as their source protein is translated and that this is enhanced in professional APCs, adding fuel to the idea that there is a specialized set of ribosomes associated with antigen presentation (*68*). This association with translation is perhaps why viral protein abundance drives neither epitope presentation levels nor T cell responses. We show that epitope presentation levels are a moderate correlate of the size of CD8^+^ T cell responses and why this has been such a slippery association to nail down. Last, we highlight the importance of measuring epitope presentation ex vivo from infected tissues and show that the technology to achieve these measurements is no longer out of reach.

## MATERIALS AND METHODS

### Study design

Data for Figs. 1 to 3 and figs. S1 to S9 came from experiments where cells were infected with VACV in vitro and samples taken at two hourly times from 0.5 to 8.5 hours for analysis by MS. Each sample was used to quantify the levels of 45 previously described VACV epitopes presented on MHC I and to determine the proteome (host cell and virus). These data were also used in Fig. 6 (D to I) and fig. S11.

Data for CD8^+^ T cell responses after intraperitoneal infection of mice with VACV that were used in correlations between epitope presentation and the size of T cell responses (Figs. 4 and 6, B, C and I) were from Dataset 1 in Croft *et al.* (*15*). These data were also used in figs. S9B and S11.

The design of experiments to quantify epitope presentation and CD8^+^ T cell responses in the spleens of mice after intravenous infection with VACV is shown in Fig. 5A. These data are used for Figs. 5 and 6 and figs. S10 and S11.

### Mice

All mice were obtained, housed, and bred from the Australian Phenomics Facility (Canberra, Australia). Mouse were housed, and all experiments were conducted according to the ethical requirements under approvals A206/45, A2020/01, and A2023/09 from the Australian National University Animal Ethics and Experimental Committee. Specific pathogen-free female C57BL/6 mice over 8 weeks of age were used for all experiments except where indicated.

### Cell lines

All immortalized cell lines, including DC2.4 and MC57G cells were cultured in T175 or T75 flasks containing Dulbecco’s modified Eagle’s medium (DMEM) + 10% fetal calf serum (FCS) + l-glutamine (D10) and passed every 3 to 4 days using trypsin to detach adherent cells. Cells were grown in incubators at 37°C in 5% CO_2_.

### Culturing primary BMDCs

BMDCs were prepared and cultured over 6 days from female mouse bone marrow cells as described by Madaan *et al.* (*69*). Female C57BL/6 mice were culled by CO_2_ asphyxiation. The femur was isolated and further cleaned with 70% ethanol. The femur was cut above the knee joint and below the epiphysis, and the contents of the femur were flushed with 2 ml of phosphate-buffered saline (PBS) using a 1-ml insulin syringe (25-gauge × ½ needle) and collected in a 50-ml centrifuge tube.

Bone marrow cells were suspended in 20 ml of cold PBS and centrifuged for 8 min (250*g*). Supernatant was removed, and cells were washed twice with PBS and resuspended in BMDC medium [DMEM; high glucose, 2 mM l-glutamine, 10% FCS, 1% penicillin/streptomycin, 55 μM 2-mercaptoethanol, and recombinant granulocyte-macrophage colony-stimulating factor (GM-CSF) (50 ng/ml)]. Eight milliliters of cells containing 10^7^ cells were added to a 90-mm petri dish (non–tissue culture grade). Four milliliters of BMDC medium was replaced on days 2, 4, and 6. Petri dishes were placed in an incubator at 37°C, 5% CO_2_ and 95% humidity for 3 days. On day 8, lipopolysaccharide was added to culture dishes to a final concentration of 1 μg/ml, incubated for 2 hours, and then cells were harvested for infection with VACV. GM-CSF (50 ng/ml) was maintained in all media, including throughout virus infection.

### Culturing PMFs

To generate primary mouse fibroblasts, 3- to 5-day-old male and female neonatal C57BL/6 mice were culled by decapitation, tails were removed, and bodies were skinned. The tails and skin were placed in a 90-mm petri dish with 4 ml of warm PBS. The skin was further finely cut with a scalpel blade (Swann-Morton) and split into two 15-ml Falcon tubes. Collagenase IV (5 ml; Worthington) was added per tube and incubated for 25 min at 37°C. Samples were centrifuged for 5 min at room temperature at 205*g*, and the supernatant was removed. Cells were washed in 5 ml of PBS and centrifuged again before the supernatant was removed. Cells were incubated in 5 ml of 0.05% trypsin for 20 min at 37°C and centrifuged again. The cell pellet was resuspended in 5 ml of fibroblast medium [DMEM, 10% FCS, 1% (v/v) MEM NEAA, 1% (v/v) penicillin streptomycin, and 1% (v/v) l-glutamine] and transferred to a single Nuclon Delta–coated, filter-capped 175-cm^2^ tissue culture flask (Thermo Fisher Scientific). Additional fibroblast medium (20 ml) was added to each flask and incubated at 37°C in 5% CO_2_. For the first four consecutive days, the medium was carefully replaced.

On day 4, cells were washed in PBS and treated with 0.05% trypsin for 3 to 5 min. Trypsin was inactivated with 10 ml of fibroblast medium, and cells were separated into four 175-cm^2^ tissue culture flasks with an additional 20 ml of fibroblast medium. Once cells reached confluency, cells were washed, trypsinized, and seeded into eight 175-cm^2^ flasks. After this step, cells were grown to confluency and split 1 in 2 over two passages to generate 32 T175 flasks.

### Growth of VACV stocks

VACV strain WR [equivalent to American Type Culture Collection VR-1354 = WR (NIH TC-adapted); GenBank: AY243312.1] was sourced directly from B Moss (NIH) and is referred to throughout as VACV. VACV was grown in BSC-1 cells grown to confluency in D10 medium. Each flask was inoculated with 3 × 10^6^ plaque-forming units (PFU) VACV in DMEM + 2% FCS + l-glutamine (D2). Flasks were incubated for 3 days at 37°C. Cells were harvested and lysed using a glass homogenizer (Wheaton) and then rapidly frozen and thawed three times. VACV stocks were purified by sucrose cushion purification using a 36% sucrose solution.

### Titration of VACV virus

VACV was diluted in 10-fold serial dilutions before adding to six-well plates with a BSC-1 cell monolayer. After 90-min incubation at 37°C with 5% CO_2_, medium was replaced with 2 ml per well of D2 with 0.4% sodium carboxymethyl cellulose. Plates were incubated at 37°C for 3 days. Virus plaques were counted by staining the cell monolayer with a crystal violet staining solution to determine virus titer.

### Infection of mice

C57BL/6 mice were infected with VACV via intravenous injection. Mice were infected with 10^6^ PFU for experiments directly measuring T cell responses, and 10^8^ PFU to directly measure VACV pMHCI presentation in vivo. Mice were monitored daily after infection.

### Infection of cell lines

BMDC, DC2.4, PMF, or MC57G cells were grown or cultured as described. Adherent cells were detached with trypsin and resuspended in D10 medium. Cell viability and concentration were measured using trypan blue staining, and 1 × 10^8^ cells were prepared for each time point. Cells were washed in 20 ml of D0 medium and resuspended in 1 ml in a 14-ml round-bottom tube (BD). One milliliter of virus was added to the cells at a multiplicity of infection of 5, and the tubes were incubated in a 37°C shaking incubator (250 rpm for 30 min). After the initial incubation, cells were transferred to 40 ml of D2 in a 50-ml centrifuge tube and incubated at 37°C for the respective time (0, 2, 4, 6, or 8 hours). The tubes were gently rolled with a MACSmix Tube rotator. At each time point, the sample was centrifuged for 5 min (1500 rpm, 4°C) and washed with cold PBS. Next, virus was inactivated in 1 ml of PBS containing trioxsalen (1 μg/ml; Sigma-Aldrich), followed by ultraviolet (UV) irradiation (Vilber Lourmat VL-215.L 365 nM UV) for 20 min on mice. Every 5 min, the samples were mixed gently. After UV irradiation, each sample was transferred to 50 ml of PBS and centrifuged for 5 min. PBS was aspirated, and samples were rapidly frozen using a dry ice ethanol bath. Samples were stored at −80°C until further use.

### Intracellular cytokine staining for IFN-γ to establish T cell response levels

Seven days after intravenous infection, mice were euthanized, and spleens were taken for analysis as previously described by Flesch *et al.* (*44*). Briefly, 1 × 10^6^ splenocytes were plated onto round-bottom 96-well plates and incubated with a final concentration of 10^−7^ M synthetic peptide at 37°C with 5% CO_2_. After 1-hour incubation, brefeldin A (50 μg/ml) was added to a final concentration of 5 μg/ml, and plates were incubated for a further 3 hours. After incubation, the splenocytes were spun at 4°C to remove the medium and stained with α-CD8-PE (BioLegend, clone 53.67) in a total volume of 50 μl per well, washed twice with fetal bovine serum (FBS)–PBS, and then fixed with 1% paraformaldehyde. Cells were centrifuged, and the medium was removed. α-IFN-γ-APC (BioLegend, clone XMG1.2) was diluted in FBS-PBS containing 0.25% (w/v) saponin. Fifty microliters was added to each well, and plates were incubated overnight at 4°C. Last, cells were washed in FBS-PBS three times and analyzed by flow cytometry.

To determine the size of a peptide-specific CD8^+^ T cell responses, or the proportion of effector CD8^+^ T cells that were immunogenic, cells were analyzed using a BD LSRII flow cytometer. For each sample, at least 50,000 single, CD8^+^ T cells were identified by gating for live singlet lymphocytes on forward scatter (FSC-A) × side scatter (SSC-A), followed by an FSC-A × FSC-H and SSC-H × SSC-W singlet gating strategy and CD8^+^ × IFN-γ to identify the proportion of CD8^+^ T cells that were IFN-γ^+^.

For each assay, eight negative controls in which no peptide was added to the well [medium with the same dimethyl sulfoxide (DMSO) concentration as peptides] were included. These were used to establish the threshold for positivity, which was defined as the mean plus 3 standard deviations of the percent of CD8^+^ T cells that were IFN-γ^+^. T cell responses were the average of positive values minus the mean background (established with negative controls).

### Solubilizing lyophilized peptide stocks

Isotopically labeled AQUA peptides were solubilized in 100% DMSO to a final concentration of 5 mM. The concentration of peptide was measured using a Direct Detect spectrophotometer (EMD Millipore) as per the manufacturer’s instructions. Peptides were diluted to 5 pmol/μl in MS buffer A.

### Cross-linking immunoaffinity columns

To prepare immunoaffinity columns for MHC I purification, 10 ml of 10% (v/v) acetic acid was incubated in poly-prep chromatography columns (Bio-Rad) for 20 min. Each column was washed with 10% (v/v) acetic acid and rinsed with Milli-Q water before drying. Protein A sepharose was added as 50% slurry to 20% ethanol, mixed, and transferred to a column. Each column was rinsed with 10 column volumes of PBS. Antibody [Y-3, specific for H-2K^b^ (*70*); 28-14-8S, specific for H-2D^b^ (*71*)] to capture MHC I complexes was diluted in PBS, added to resin at a concentration of 10 mg per 1 ml of resin, and then incubated for 1 hour at 4°C while gently rotating, before transferring back to the column. Ten column volumes of borate buffer (50 mM boric acid, 5 0 mM KCl, and 3.97 mM NaOH) were used to wash the unbound antibody, followed by 10 column volumes of 0.2 M triethanolamine (pH 8.0). Bound antibody was cross-linked to protein A sepharose using 40 mM dimethyl pimelimidate cross-linker for 1 hour at room temperature. Cross-linking was stopped by the addition of cold 0.2 M tris (pH 8). Each column was rinsed with 10 column volumes of 0.1 M citrate buffer (0.1 M citrate, pH 3.0), followed by 10 column volumes of borate buffer. Prepared columns were stored at 4°C before use.

### Homogenization of infected cells

To homogenize infected cells and spleens for pMHCI immunopurification, spleens were snap frozen, ground in a Retsch Mixer Mill MM 400 under cryogenic conditions, and resuspended in a solution to deactivate and solubilize pMHCI using a lysis buffer containing 0.5% IGEPAL CA-630 (Sigma-Aldrich), 50 nM tris-HCl (pH 8.0), 150 mM NaCl, and a complete protease inhibitor tablet (Roche Molecular Biochemicals).

### Immunoaffinity purification of pMHCI complexes

Purification of pMHCI columns were modeled on the protocol described by Purcell *et al.* (*72*). Cross-linked antibody and protein A sepharose slurry (1.5 mg) was added to a clear poly-prep column. Each column was washed with 10 column volumes of wash buffer 1. Cell lysate was added to a precolumn, containing protein A sepharose that did not contain cross-linked antibody, to remove contaminants binding nonspecifically to protein A. Next, flow-through from the precolumn was dripped directly onto an immunoaffinity column containing α-H-2K^b^ antibody bound to protein A sepharose. To ensure efficient binding of pMHCI to the antibody, the lysate was mixed and incubated for 5 min at 4°C. The lysate passed through the column by gravity flow and added to the same column to pass the lysate again. Flow-through was added directly onto an α-H-2D^b^ column, mixed with the cross-linked protein A sepharose slurry, and passed through the column as described for H-2K^b^. Each immunoaffinity column was washed to remove unbound material and contaminants sequentially with buffer 1 [0.005% (v/v) IGEPAL, 50 mM tris, 150 mM NaCl, 5 mM EDTA, 100 μM phenylmethylsulfonyl fluoride, and pepstatin(1 μg/ml)], buffer 2 (50 mM tris and 150 mM NaCl), buffer 3 (50 mM tris and 450 mM NaCl), and buffer 4 (50 mM tris). Antibody-capture pMHCI was eluted with 5 column volumes of 10% (v/v) acetic acid from each column separately. Last, 50 fmol of AQUA peptide was added to the eluate.

### High-performance liquid chromatography purification and fractionation

Eluate from immunoaffinity purification contained peptides, the MHC I heavy chain, and β2-microglobulin. To separate these molecules, the eluate was fractionated as described by Croft *et al.* (*30*) using reverse-phase high-performance liquid chromatography (RP-HPLC) on a 4.6-mm internal diameter by 50-mm long RP C18 HPLC column (Chromolith Speed Rod, Merck) using an AKTAmicro HPLC system (GE Healthcare). HPLC buffer A [0.1% trifluoroacetic acid (TFA) and 99% Optima MS-grade water] and HPLC buffer B (0.1% TFA, 80% acetonitrile, and 19.9% Optima water) were used in the phase buffer system with a flow rate of 1 ml/min. To separate peptides from the MHC I heavy chain and β2-microglobulin, and fractionate the peptides by hydrophobicity, peptides were separated across a gradient of HPLC buffer B from 2 to 45% over 20 min. Five microliters of each peptide fraction was collected in LoBind Eppendorf tubes and concentrated by vacuum evaporation. Peptide fractions were pooled using a concatenation strategy, maximizing retention time distribution across the subsequent RP-HPLC used during MS detection.

### Preparation of cell lysate for proteomics analysis

Flow-through from each immunoprecipitation experiment was kept for proteomics analysis. Approximately 50 μg of lysate material was treated with the reducing agent tris(2-carboxyethyl)phosphine (TCEP) at a final concentration of 5 mM and heated to 60°C for 30 min before being loaded onto a Filter-Aided Sample Preparation (FASP) protein digestion column (Abcam) (*73*). Detergent was removed from the sample by washing twice with 8 M urea in 100 mM tris-HCl (pH 8) and centrifuged at 15,000 relative centrifugal force (RCF) for 15 min at room temperature. Cysteines were alkylated by addition of 50 mM iodoacetamide for 20 min in the dark and were washed three times with 8 M urea and three times with 50 mM ammonium bicarbonate. Proteins were subjected to tryptic digestion (1:100, w/w) overnight at 37°C. Tryptic peptides were collected by centrifugation and washing of the column with 40 μl of 50 mM ammonium bicarbonate and 50 μl of 500 mM sodium chloride. Eluted tryptic peptides were acidified with 1% (v/v) formic acid and desalted with C_18_ Omix tips (Agilent).

### Analysis of peptides by MRM MS

Following MHC-peptide elution, samples were concentrated to a volume <10 μl using a Labconco Centrivac concentrator at 40°C, and the volumes were equalized to 20 μl in 0.1% formic acid in Milli-Q water. Samples were sonicated in a water bath for 10 min, centrifuged for 10 min at 15,000 RCF, and stored in MS vials at 4°C for immediate analysis. Peptide abundance was measured as described by Croft *et al.* (*30*). Using a SCIEX QTRAP 5500+ MS coupled with an on-line Eksigent Ekspert nanoLC 415 (SCIEX, Toronto, Canada). Ten microliters of each sample was directly loaded onto a trap column [ChromXP C_18_, 3 μm 120°A, 350 μm by 0.5 mm (SCIEX)], maintained at an isocratic flow of buffer A at a flow rate of 5 μl/min. Peptides were separated using an analytical column [ChromXP C_18_, 3 μm 120°A, 75 μm by 15 cm (SCIEX)] by increasing linear concentrations of buffer B at a flow rate of 300 nl/min for 75 min. In MRM mode, unit resolution was used for Q1 and Q3, coupled to an information-dependent acquisition criterion triggering an Enhanced Product Ion scan (10,000 Da/s; rolling CE; unit resolution) following any MRM transition exceeding 100 counts and ignoring the triggering MRM transition for 3 s thereafter. For analysis of peptides presented ex vivo from splenocytes, the same conditions were used except that samples were acquired on a SCIEX QTRAP 6500+ MS.

### LFQ of protein expression

Following desalting of tryptic peptides, the equivalent of ~2 μg of material was analyzed on a Q Exactive Plus Orbitrap mass spectrometer (Thermo Fisher Scientific) coupled online to an Ultimate 3000 nanoLC system (Thermo Fisher Scientific). Samples were loaded onto an Acclaim PepMap100 trap column (100 μm by 2 cm, nanoViper C_18_, 100 Å pore size; Thermo Fisher Scientifc) at a flow rate of 15 μl/min in 2% (v/v) acetonitrile and 0.1% (v/v) formic acid in water. Peptides were eluted across a PepMap100 C_18_ nano column (75 μm by 50 cm, 3 μm, 100 Å pore size; Thermo Fisher Scientific) at 250 nl/min with buffer A using a gradient of buffer B as follows: hold at 2.5% for 2 min, ramp to 7.5% for 1 min, then add 40% buffer over the course of 120 min, with a subsequent ramp to 99% over 5 min before equilibrating back to 2.5% over 1 min and maintaining this for 20 min to end the run. The instrument was operated in DDA mode with the following settings: full MS scan with a mass range of 375 to 1800 mass/charge ratio (*m*/*z*), resolution of 70,000 at 200 *m*/*z*, automatic gain control (AGC) of 3 × 10^6^, and maximum ion injection time of 50 ms, with dynamic exclusion set to 20 s. The top 12 most intense ions were selected for Higher-Energy Collisional Dissociation fragmentation. A normalized collision energy of 27 was used, with maximum injection time of 120 ms, 1.8 *m*/*z* isolation window, 17,500 resolution, and AGC of 2 × 10^5^.

Data were analyzed by LFQ using MaxQuant (1.5.2.8) (*74*) with a combined database of the reference *Mus musculus* proteome (2016, UniProt) appended with the Vaccinia Virus Western Reserve strain reference proteome. Carbamidomethyl (C) was set as a fixed modification and with acetyl (protein N terminus) and oxidation (M) set as variable modifications. MS/MS tolerance was set to 20 parts per million, with peptide spectrum match, protein and site false discovery rate set to 1%. Trypsin was used as the enzyme, with a minimum peptide length of 7 and maximum peptide length of 40. “Match between runs” and “Include contaminants” were set to true. The resulting protein output was analyzed by Perseus (1.6.0.7). Proteins identified as “only by site,” “reverse,” or “contaminants” were removed, and LFQ intensities were log_10_ transformed. Missing data were subjected to imputation using the default Perseus method.

### Calculation of peptide abundance

The area for each MRM transition was calculated by Skyline Targeted Mass Spec Environment application (MacCoss Lab Software) (*75*). The area of the transitions for each peptide was added together to calculate the total peak area. For peptides with internal fragment transitions that could not be incorporated into Skyline, quantification was carried out by extracting the peak area under the curve using PeakView (SCIEX).

To estimate the amount of peptide recovered from each spleen, the total peak area, the ratio of the total peak area of the endogenous peptide with the total peak area of the AQUA peptide, was obtained. The total amount of endogenous peptide recovered was calculated by multiplying by the amount of AQUA peptide added to the sample (50 fmol). The equation is summarized below

$$\begin{array}{c} \text{Peptide recovered}=\text{total peak area}\;\left(\text{endogenous}\right)\\ /\text{total peak area}\;\left(\text{AQUA}\right)\times \text{mol}\;\left(\text{AQUA}\right) \end{array}$$

Epitope abundance and relative protein abundance were measured from two independent experiments.

### Statistical analysis

Average or mean refers to the arithmetic mean to estimate epitope abundance, and geometric mean to compare relative abundance of protein estimated by LFQ. PCAs for mouse, VACV, or both mouse and VACV protein relative abundance were derived from log-transformed LFQ values from each replicate using the base stats (4.0.3) in R using RStudio (*76*–*78*). Variables were centered but not scaled. The Perseus method described by Tyanova *et al.* (*79*) was used as an imputation strategy for undetected LFQ values on the basis that these undetected proteins were assumed to occur at very low levels. Briefly, for each sample containing both mouse and VACV proteins, data were log transformed, and the median and standard deviation of the log(LFQ) values were calculated for non-null LFQ values. Null LFQ data were imputed by a random distribution–centered 1.8 standard deviations below the LFQ detected median, with a distribution of 0.5 × standard deviation. For the geometric mean plots in Fig. 2, null values for host proteins were given an arbitrarily low value (10^3^ intensity).

Pairwise Spearman/Pearson correlations were calculated in Python (*80*–*83*) on log-transformed values. Nonimmunogenic T cell responses were given an arbitrarily low value of 0.01%. For comparisons involving pMHCI affinity, individual epitopes were excluded from analysis where measured pMHCI affinity was not available. VACV protein kinetic class (E1.1, E1.2, I, L) for each protein was derived from the following publications based on VACV WR open reading frame nomenclature (*42*, *43*).
