# Supplementary material for: A poxvirus model reveals general correlates of antigen presentation and immunogenicity for viral CD8+ T cell epitopes
Source: Sci Adv. 2025 Dec 19;11(51):eaea8105. doi: 10.1126/sciadv.aea8105 (PMC12716421; doi:10.1126/sciadv.aea8105)
Supplement: Supplementary file 1 — Figs. S1 to S12 Legend for data S1 Table S1 [file sciadv.aea8105_sm.pdf]

Science Advances
AAAS

Supplementary Materials for

A poxvirus model reveals general correlates of antigen presentation and immunogenicity for viral $CD8^+$ T cell epitopes

Matthew J. Witney *et al.*

Corresponding author: David C. Tscharke, david.tscharke@anu.edu.au

Sci. Adv. **11**, eaea8105 (2025)
DOI: 10.1126/sciadv.aea8105

The PDF file includes:

Figs. S1 to S12
Legend for data S1
Table S1

Other Supplementary Material for this manuscript includes the following:

Data S1

Fig. S1.

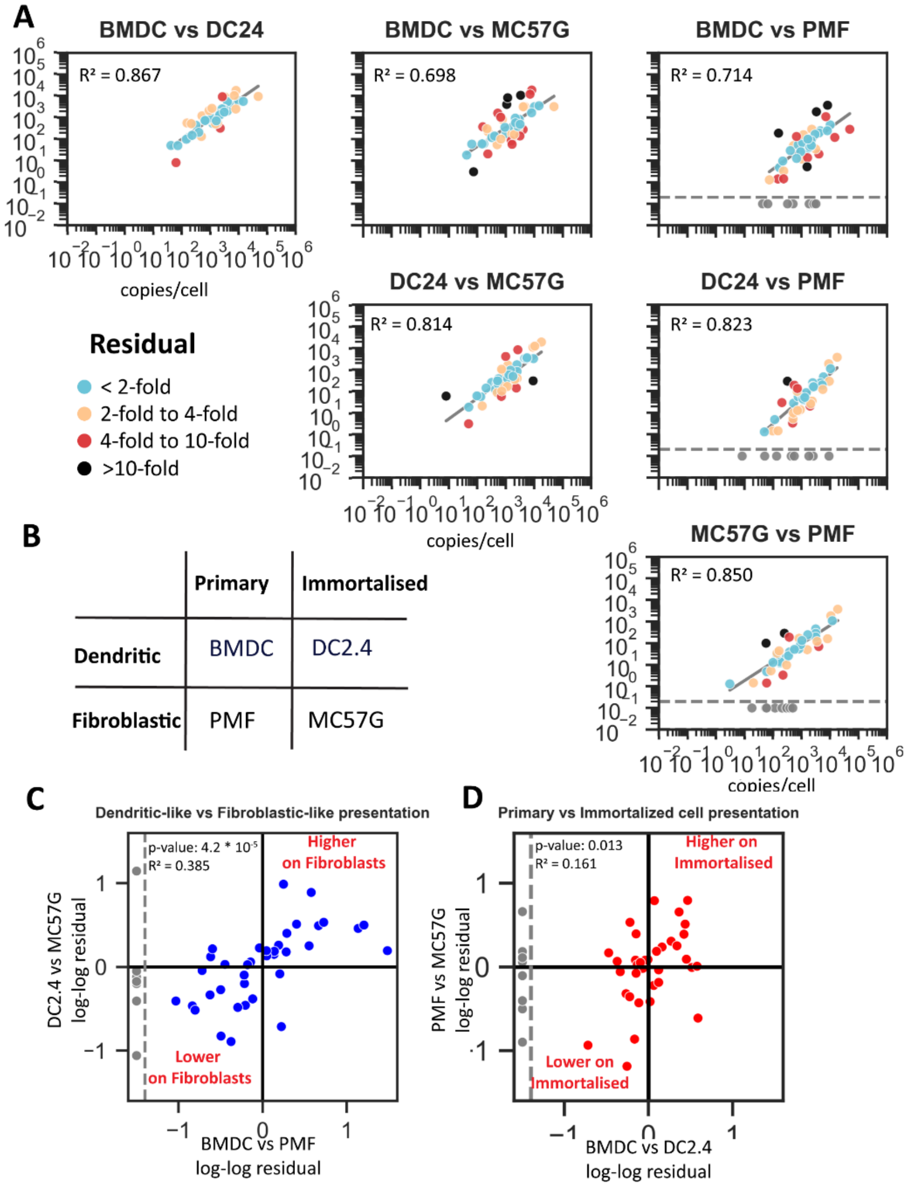

Pairwise comparisons of VACV epitopes abundance on different cell types. BMDC, DC2.4, MC57G and PMF cells were infected with VACV. p:MHC-I abundance of 45 epitopes were measured by MRM at 0.5, 2.5, 4.5, 6.5 and 8.5h post infection in two independent experiments. The sum of the average abundance for each epitope at all timepoints were compared between different cell lines. A) Pairwise correlation of p:MHC-I abundance measured on cell types. Statistics calculated using Pearson correlation from log-converted values. Diagonal lines represent line of best fit. Points are colour coded by distance from the line of best fit as indicated. Undetected p:MHC-I are shown below the horizontal dashed line but excluded from statistical analysis. B) Summary of shared features between BMDC, DC2.4, MC57G and PMF cells. C-D) Correlation of residuals f from pairwise comparisons of BMDC v PMF and DC2.4 v MC57G (C) or BMDC v DC2.4 and PMF v MC57G (D).

Fig. S2.

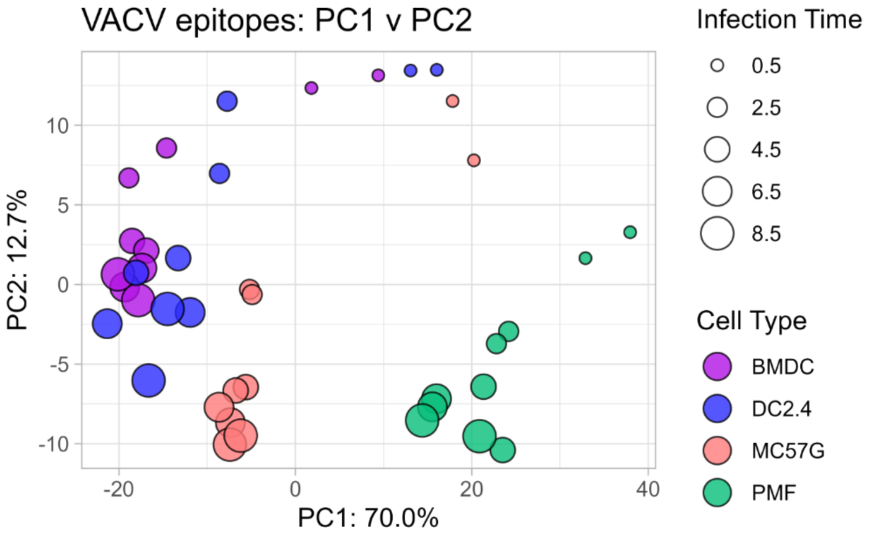

PCA plots for epitope datasets.

PCA plots describing PC1 and PC2 axis for VACV p:MHC-I abundance for each replicate and timepoint on VACV-infected BMDC, DC2.4, MC57G and PMF cell types. Colours indicate cell type, the size of each point identifies the time after infection.

Fig. S3.

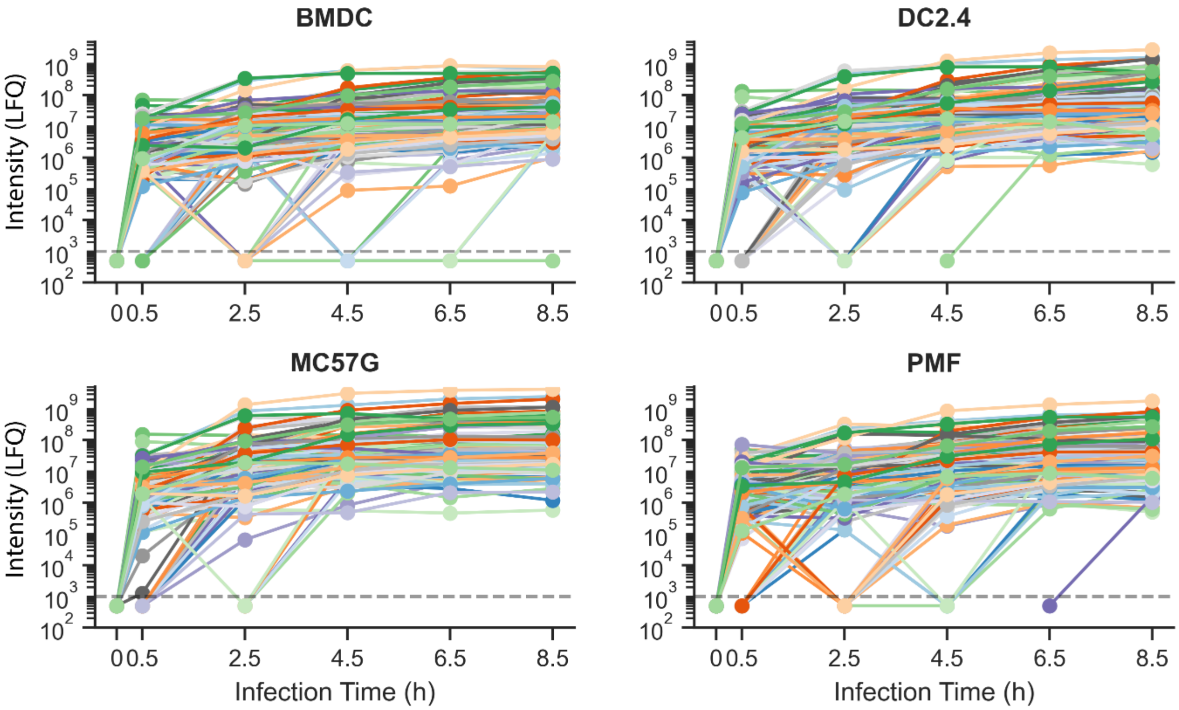

Kinetics of VACV proteome expression across four cell types. BMDC, DC2.4, MC57G and PMF cells were infected with VACV between 0.5h and 8.5hours. Protein abundance was estimated by label-free quantification (LFQ). The plots for cell type as marked show the average abundance of VACV proteins at each time. Points below the dotted line represent undetected proteins. Lines are drawn from the time-point before first detection.

Fig. S4.

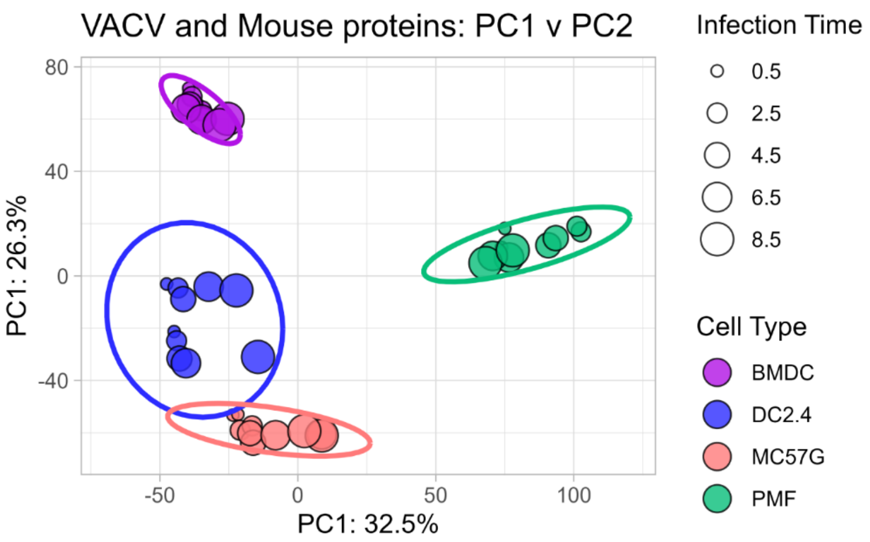

PCA plots for complete protein datasets. PCA plots describing PC1 and PC2 axis for combined mouse and VACV protein LFQ intensity for each replicate and timepoint on VACV-infected BMDC, DC2.4, MC57G and PMF cell types. Colours indicate cell type, the size of each point identifies the time after infection.

Fig. S5.

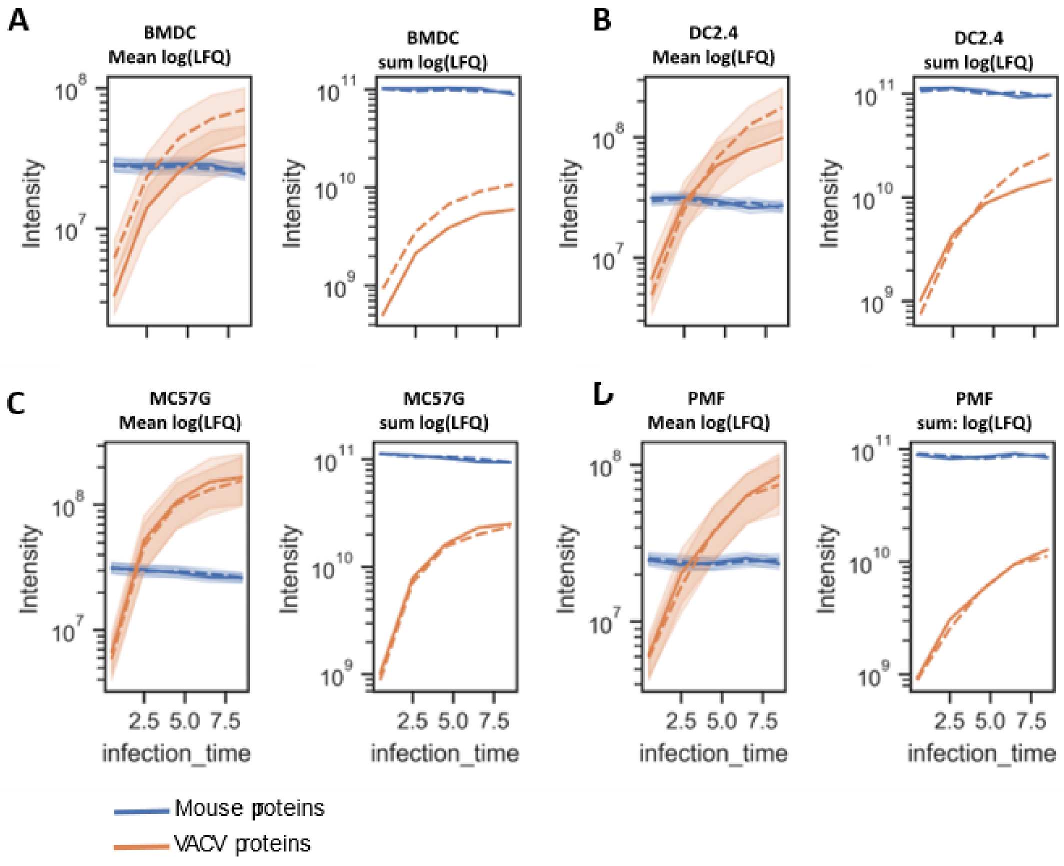

Relative protein abundances of VACV and mouse proteins over time. Each plot compares the geometric mean or the sum of the relative abundance of mouse (blue) and VACV (orange) proteins on BMDC (A), DC2.4 (B), MC57G (C) and PMF cells (D) over time. Each line represents a separate replicate. Shaded areas represent the variation in abundance of individual proteins.

Fig. S6.

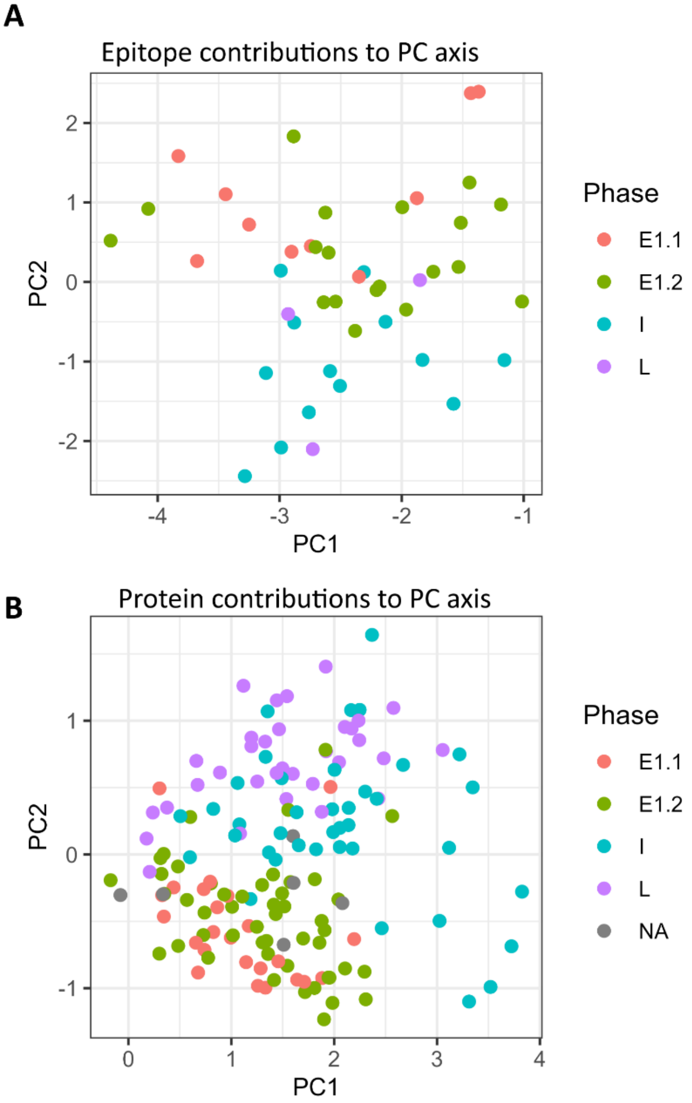

Variable contributions towards PC1 and PC2 axes. The relative contributions of variables towards PC1 and PC2 axes from the PCA analysis of either VACV epitopes (A) or VACV proteins (B) as shown in Fig. 1 and Fig. 2 respectively. Each point identifies the loading of each epitope or protein contributing to PC1 and PC2, multiplied by the size of the PC eigenvalue. Points are coloured by the kinetic class (E1.1, E1.2, Intermediate (I), Late (L)) of the respective VACV protein.

Fig. S7.

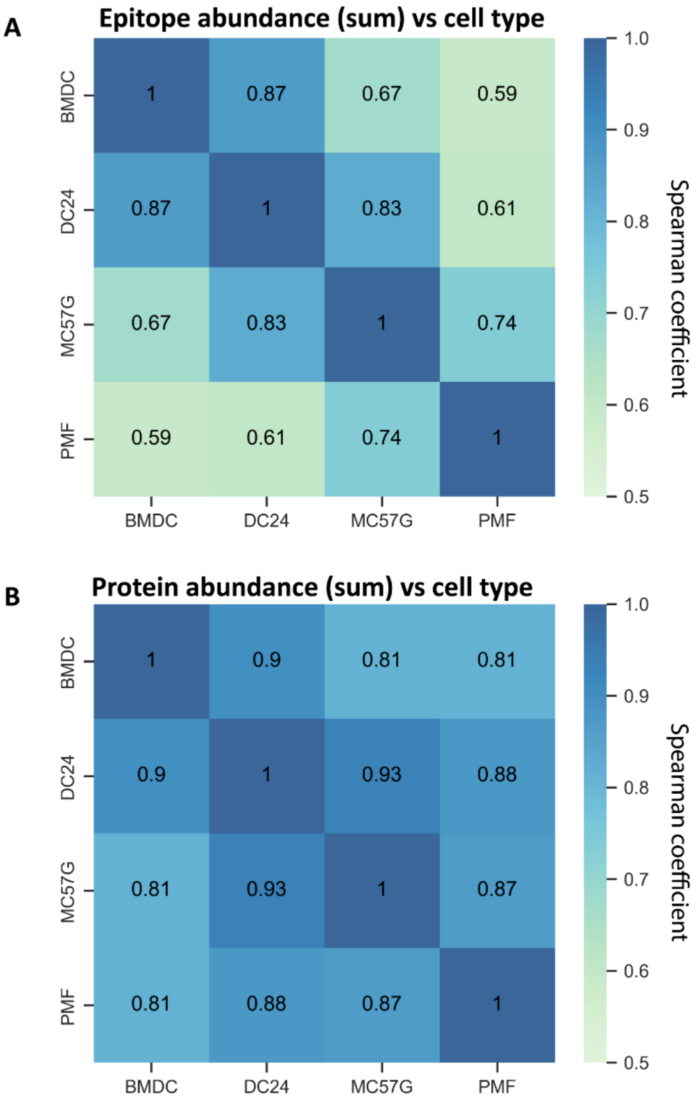

Conservation of p:MHC-I kinetic class between cell types. Spearman correlation coefficients for pairwise comparisons between epitope (A) and protein (B) abundance measured on each cell type. The abundance of each epitope and protein was evaluated as the averaged sum of values across all timepoints on each cell type.

Fig. S8.

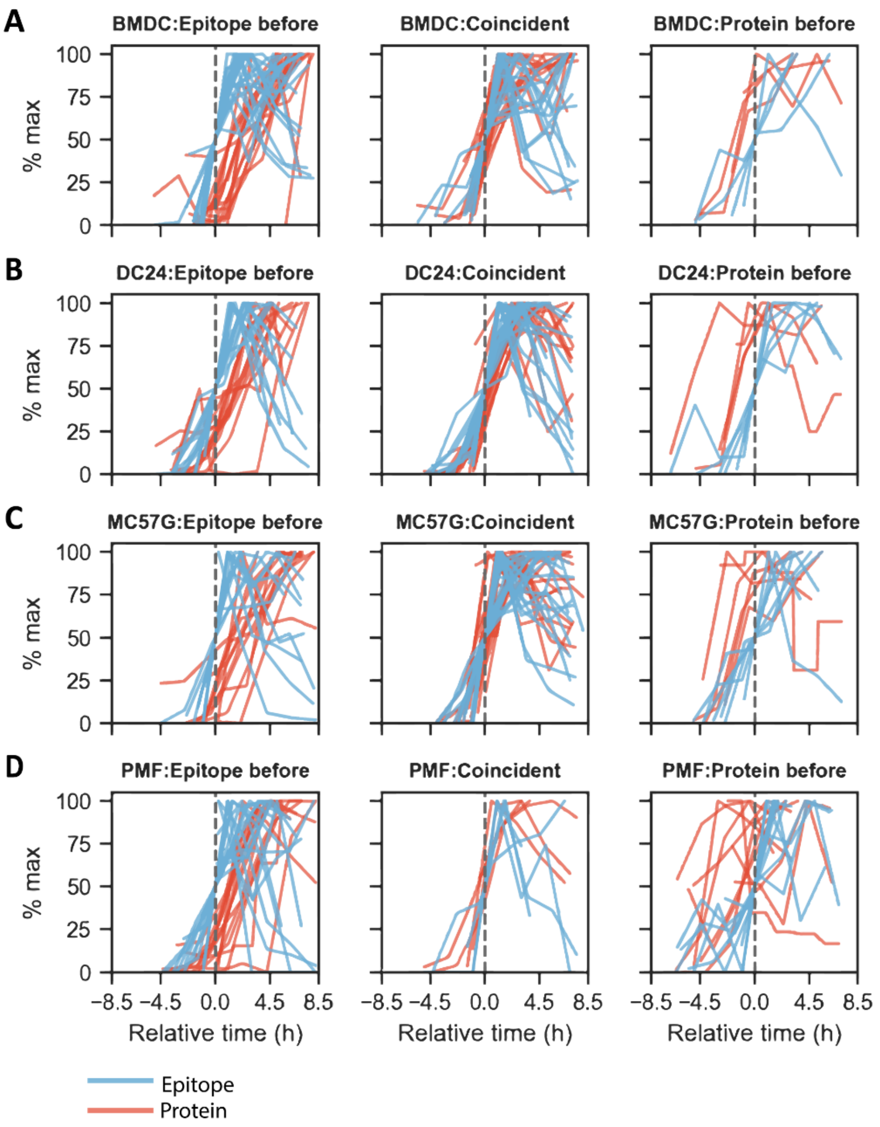

VACV epitope presentation kinetics, classified as before, coincident or after protein expression. The average p:MHC-I abundance (MRM MS) and VACV protein abundance (LFQ) from two replicates were normalised with respect to the maximum value over the time-course. Each epitope was matched to the corresponding VACV antigen. The time to 50% of the maximum value (tmax/2) for epitope and protein abundance was calculated on VACV-infected BMDC (A), DC2.4 (B), MC57G (C) and PMF cells (D). Epitope presentation before, coincident with, or after protein expression was classified according to whether the epitope tmax/2 was at least 1 hour earlier, less than one hour, or more than one hour after the tmax/2 of the respective protein. Relative time represents protein and epitope abundance translated along the x-axis such that epitope tmax/2 is centred at 0. Blue lines and red lines identify average kinetics of each epitope and protein respectively.

Fig. S9.

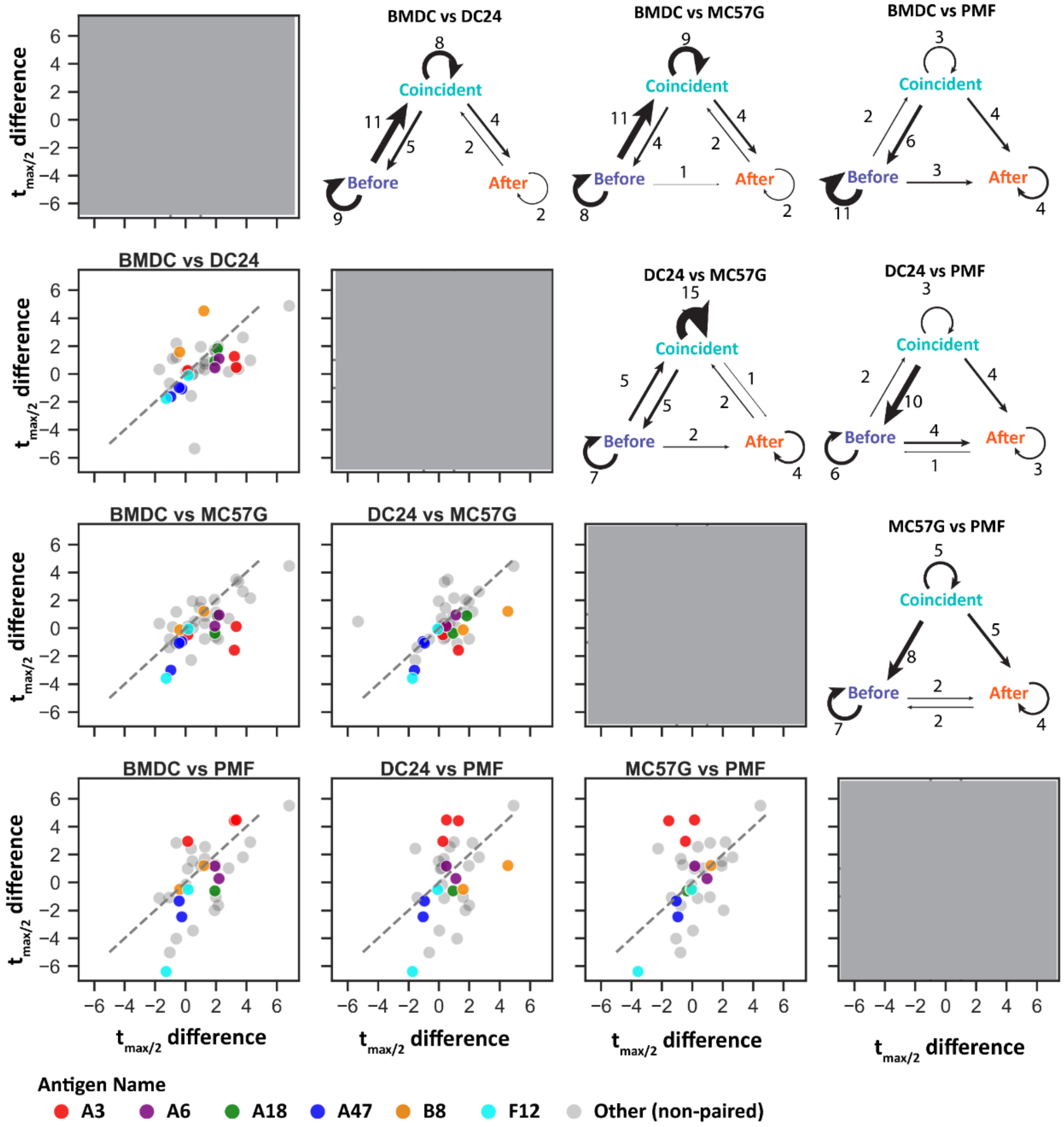

Conservation of p:MHC-I kinetic class between cell types. Upper right: Arrows indicate the number of p:MHC-I that were redefined into new classification between the two respective cell types. Size of arrows are scaled to the number indicated. Lower left: Pairwise comparisons describing the difference between the tmax/2 of the protein and epitope on the respective cell types. p:MHC-I derived from the same VACV protein are coloured similarly as indicated. Unrelated p:MHC-I shown in grey. The diagonal dashed line represents position expected for p:MHC-I with the same time gap between epitope and protein tmax/2 for the two cell types.

Fig. S10.

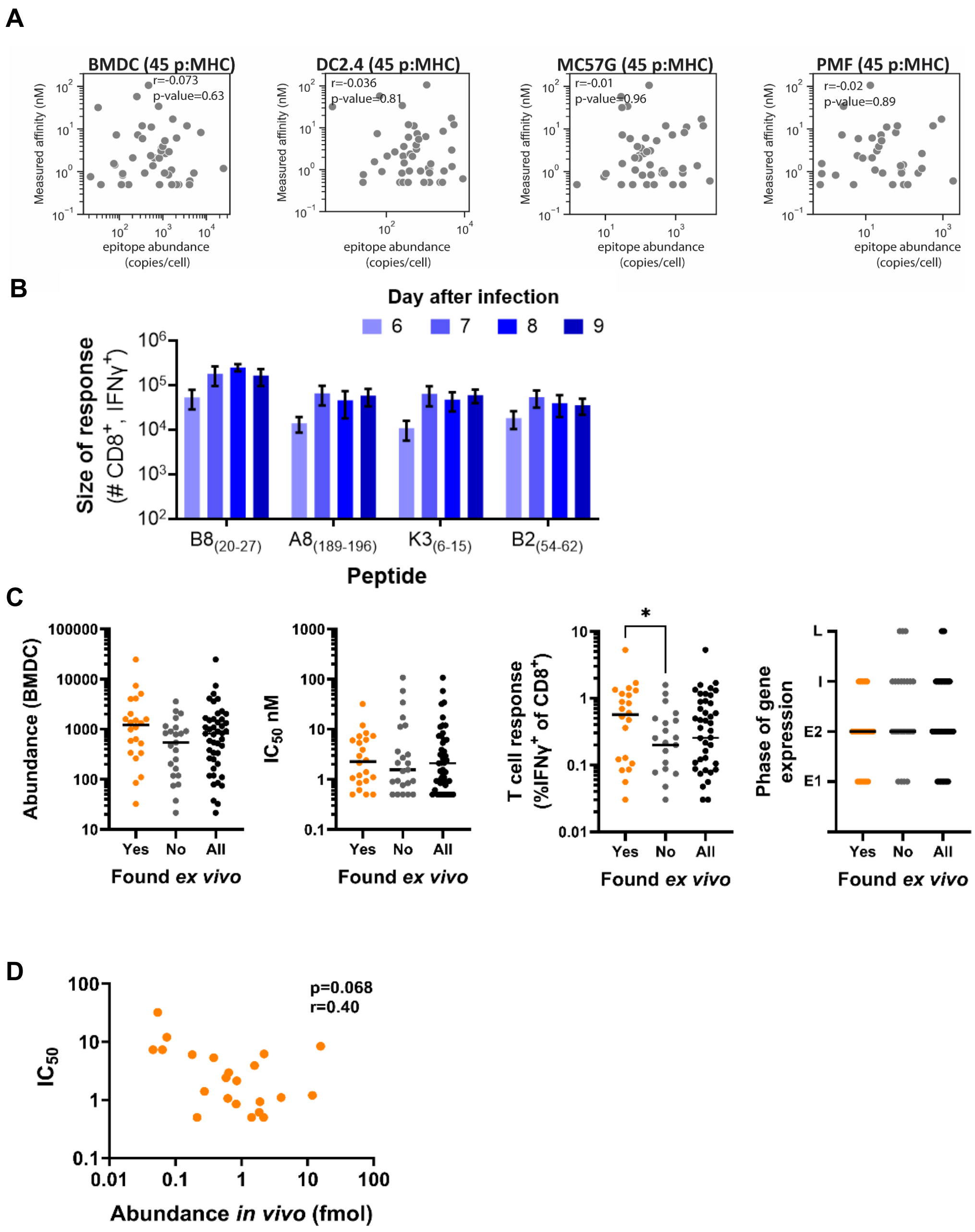

Properties of VACV epitopes detected *in vitro* and *in vivo*. Properties A) Correlation of measured affinities (PMID: 30718433) and epitope abundance measured on 4 cell types *in vitro*. Statistic: spearman correlation. B) Size of $CD8^+$ T cell responses for VACV epitopes on days 6-9 after intraperitoneal infection of mice. C) Comparison of variables, as shown on Y-axis, for epitopes found *in vivo* compared with those not found *in vivo* and all 45 epitopes in the study. Statistical testing was done between all pairs for Yes and No, and Yes and All groups of epitopes using Mann-Whitney tests, only significant where shown; *p<0.05. D) Correlation of measured affinity and epitope abundance measured *in vivo*. Statistic: spearman correlation.

Fig. S11.

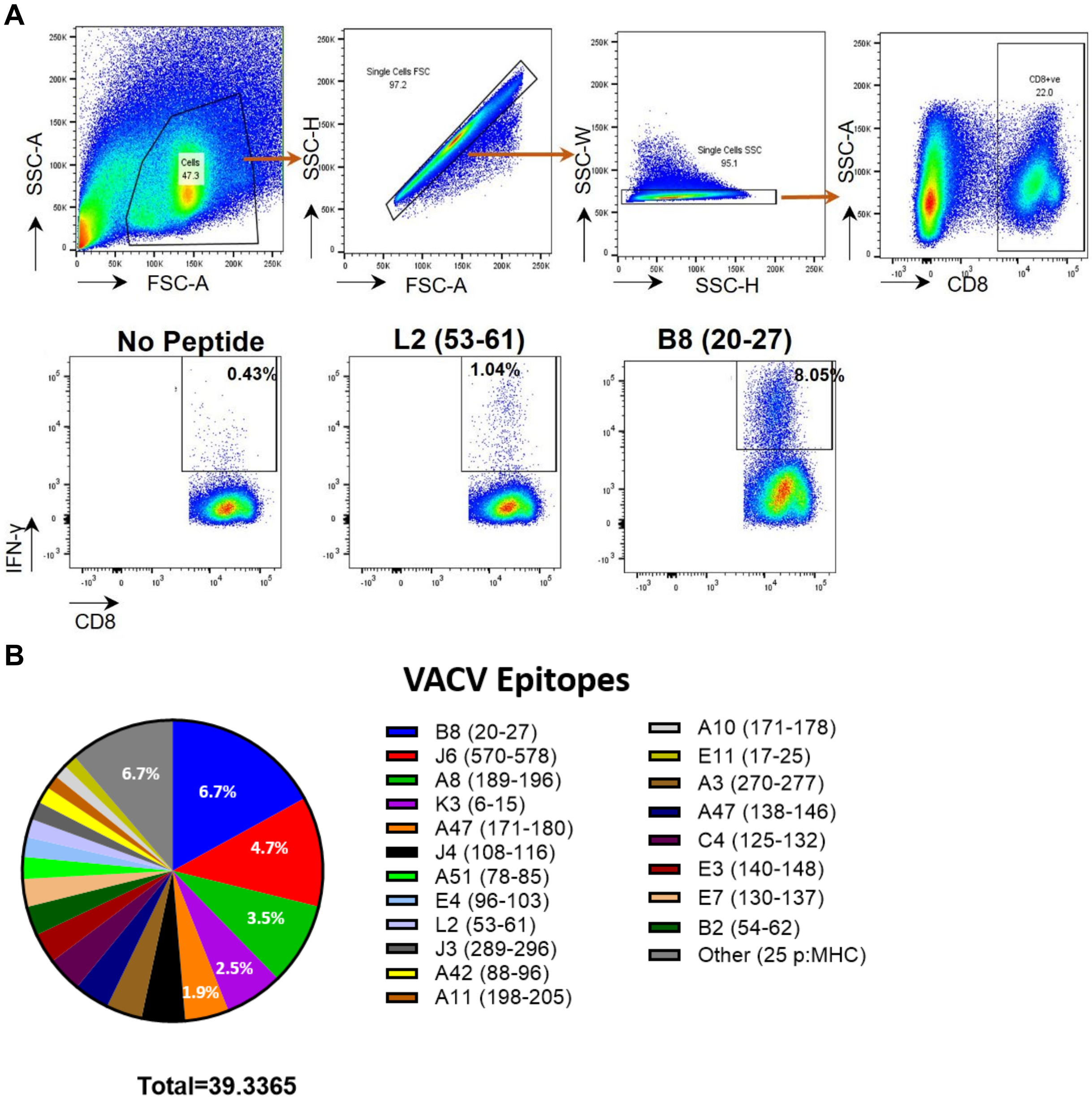

CD8⁺ T cell responses to VACV epitopes after intravenous infection. (**A**) Representative gating of flow cytometry plots. (**B**) Each peptide-specific T cell response is represented by a coloured fraction of the total IFNγ$^+$ T cell response that could be accounted for by the 45 epitopes investigated in this study. Numbers in segments are the average % of CD8$^+$ T cells that make IFNγ$^+$ after *ex vivo* stimulation of splenocytes seven days after intravenous infection of mice with VACV.

Fig. S12.

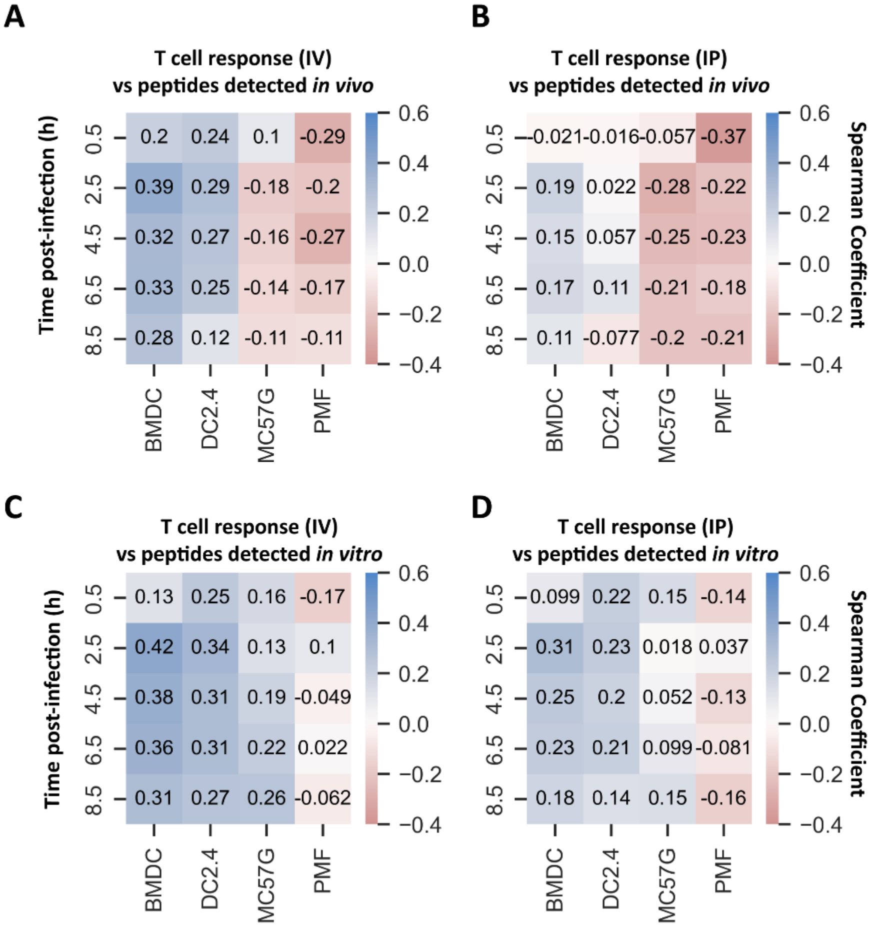

Exhaustive pairwise comparisons of p:MHC-I abundance and T cell responses. The spearman correlation coefficients comparing epitope abundance on each cell type and time-point post infection with peptide-specific T cell responses following intravenous (A,C) or intraperitoneal (B,D) infection and in C57BL/6 mice. Comparisons were made either restricted to the set of peptides detected *in vivo* (A,B) or the complete set of 45 epitopes (C,D). Epitope abundances not detected in a given timepoint were given an arbitrarily low value of 0.01 copies/cell.

Data S1. (separate file)
Excel file that includes a summary of all data and tabs for each raw data set.

Table S1. Resources.

REAGENT or RESOURCE	SOURCE	IDENTIFIER
Antibodies		
Anti-CD8 (PE) Clone 53-6.7	Biolegend	
Anti-IFN-γ (APC) Clone XMG1.2	Biolegend	
Anti-H-2K^b Clone Y-3 hybridoma (HB-176)		(*70*)
Anti H-2D^b Clone 28-14-8S hybridoma (HB-27)		(*71*)
Bacterial and virus strains		
Vaccinia Virus Western Reserve (VACV)	Dr. Bernard Moss.	ATCC VR-1354
Chemicals, peptides, and recombinant proteins		
Recombinant Mouse GM-CSF Protein	RNDSystems	415-ML-050
Dulbecco's Modified Eagle Medium; high glucose (DMEM)	Sigma-Aldrich	D6546
Penicillin/Streptomycin	Gibco	15140-122
Dimethyl sulfoxide (DMSO)	Sigma-Aldrich	472301-100ML
Foetal bovine serum (FBS)	Sigma	F9423-500mL
Carboxymethylcellulose (CMC) sodium salt	Millipore Sigma	C4888-500G
Minimum Essential Medium Non-essential Amino Acids (MEM NEAA)	Thermo Fisher Scientific	11140076
L-glutamine	Thermo Fisher	10313039
2-mercaptoethanol	Thermo-Fisher	21985023
Phosphate Buffered Solution (PBS)	Gibco	10010023
Brefeldin A	Sigma	B7651
Saponin	Sigma	47036-50G-F
Paraformaldehyde (16%)	Electron Microscopy Sciences	15710
Lipopolysaccharide (LPS) from Escherichia Coli	Sigma-Aldrich	L4391-1MG
Trypsin-EDTA (0.5%) no phenol red	Gibco-EDTA (0.5%)	15400054
Acetic acid (ACS grade)	Sigma-Aldrich	33209-1L-GL
Trioxsalen (2.5mg/mL)	Sigma-Aldrich	T6137
Triethanolamine (0.2M, pH 8.3)	Thermo Fisher Scientitic	787-500ml
IGEPAL CA-630 (NP-40)	Sigma-Aldrich	I8896
Protein A-CaptivA PriMab 1L	RepliGen-USA	CA-PRI-1000
Tris, Ultra Pure Grade	Astral Scientific	BIO3094T
Sodium chloride	Merck Millipore	1064041000
Pepstatin A (1mg/mL)	MP Biochemicals	219536810
Phenyl methylsulfonyl fluoride (PMSF) (0.1M)	Sigma-Aldrich	P7626-1G
Trifluoracetic acid (TFA)	Thermo Fisher Scientific	IE28904

Citric acid monohydrate	Merck Millipore	1.00244.0500
Boric Acid	Astral Scientific	IOBB0044
Acetonitrile	Fisher	FL-17-0496
Pierce™ Formic Acid, LC-MS Grade	Sigma-Aldrich	14265-1ML
Complete protease inhibitor tablet	Sigma-Aldrich	11836145001
Dimethyl pimelimidate dihydrochloride (DMP)	Merck	D8388-250MG
Triton-X	Sigma-Aldrich	X100-500mL
Autosampler vials	Thermo Scientific	THC160134
EDTA	Chemsupply	326410003617
Critical commercial assays		
C18 HPLC column: 4.6-mm internal diameter × 50- or 100-mm long reversed-phase C18 end-capped HPLC column	(Chromolith Speed Rod, Merck)	1514500001 and 1021290001)
Deposited data		
MS data: ftp://MSV000095910@massive.ucsd.edu		
Username: MSV000095910_reviewer		
Password: VacciniaEpitopes		
Experimental models: Cell lines		
DC2.4	K Rock, U.Mass.	*(40)*
MC57G	J Yewdell, NIH	*(39)*
BSC1	ATCC	CCL-26
143-B TK-	J Yewdell, NIH	
Experimental models: Organisms/strains		
Mus Musculus C57BL/6		
Peptides		
See Data S1 for sequences	Genscript	>90% purity
Software and algorithms		
Python	3.10.9	
R	R04.0.3	(*77*)
RStudio	1.4.1103	(*76*)
Maxquant	1.5.2.8	(*74*)
Perseus	1.6.0.7	(*79*)
Skyline Targeted Mass Spec Environment application	21.2.0.568 (MacCoss Lab Software)	(*75*)
Flowjo Software	Tree Star, Ashland OR	
python packages:<br>pandas 1.5.3<br>seaborn 0.12.2<br>matplotlib 3.7.0<br>scipy 1.10.0<br>numpy 1.23.5<br>jupyter notebook 6.5.4		(*83*)<br>(*80-82*)

**R packages:**<br>Stats 4.0.3<br>readxl_1.4.3<br>gridExtra_2.3<br>scales_1.2.1<br>gplots_3.1.3<br>RColorBrewer_1.1-3 ggforce_0.3.3<br>ggsci_3.0.0<br>factoextra_1.0.7<br>lubridate_1.9.3<br>forcats_1.0.0<br>stringr_1.5.0<br>dplyr_1.1.3<br>purrr_1.0.2<br>readr_2.1.4<br>tidyr_1.3.0<br>tibble_3.2.1<br>ggplot2_3.4.4<br>tidyverse_2.0.0<br>fgsea_1.16.0		(*78*)
GraphPad Prism 9	GraphPad Software	
Other		
SCIEX QTRAP 6500+	SCIEX	
SCIEX QTRAP 5500	SCIEX	
Eksigent Ekspert nanoLC 415 (SCIEX, Toronto, Canada).	SCIEX	
trap column (ChromXP C18, 3 µm 120˚A, 350 µm × 0.5 mm)	SCIEX	
AKTA micoHPLC system	GE Healthcare	
Retsch Mixer Mill Multipurpose Mill 400	Retsch	
FACS Flow Cytometer LSRII	BD Biosciences	
Sonicator	Branson Sonifier 450	
Ultrasonic Disintegrator (MSE Soniprep 150)	Bioequipment Scientific	
Direct Detect®spectrophotometer	EMD Millipore	
96-well plate (U-bottom)	Corning Incorporated	3799
14 mL round bottom tube	BD	
ultraviolet (UV) irradiation (365nM UV)	Vilber Lourmat	VL-215.L
MACSmix™ Tube rotator	Miltenyi Biotec	
0.22 µM syringe filter	Minisart	16534-K
Cell strainer (50 µM Nylon)	Falcon	352340
